# Publisher Correction: Maternal preconceptional and prenatal exposure to El Niño Southern Oscillation levels and child mortality: a multi-country study

**DOI:** 10.1038/s41467-024-51533-0

**Published:** 2024-08-15

**Authors:** Hongbing Xu, Castiel Chen Zhuang, Vanessa M. Oddo, Espoir Bwenge Malembaka, Xinghou He, Qinghong Zhang, Wei Huang

**Affiliations:** 1 Department of Occupational and Environmental Health, Peking University School of Public Health, Beijing, China; 2 Peking University Institute of Environmental Medicine, Beijing, China; 3 Peking University School of Economics, Beijing, China; 4 Department of Kinesiology and Nutrition, College of Applied Health Sciences, University of Illinois Chicago, Chicago, IL USA; 5 Center for Tropical Diseases and Global Health, Université Catholique de Bukavu, Bukavu, Democratic Republic of the Congo; 6 Faculty of Medicine, Université Catholique de Bukavu, Bukavu, Democratic Republic of the Congo; 7 Department of Epidemiology, Johns Hopkins Bloomberg School of Public Health, Baltimore, MD USA; 8 Department of Atmospheric and Oceanic Sciences, School of Physics, Peking University, Beijing, China

**Keywords:** Risk factors, Environmental health, Developing world

Correction to: *Nature Communications* 10.1038/s41467-024-50467-x, published online 17 July 2024

In the original version of this Article, Figs. 2 and 3 were mistakenly interchanged. This has been corrected in both the PDF and HTML versions of the Article.

