# Supplementary material for: Maternal preconceptional and prenatal exposure to El Niño Southern Oscillation levels and child mortality: a multi-country study
Source: Nat Commun. 2024 Jul 17;15:6034. doi: 10.1038/s41467-024-50467-x (PMC11254917; doi:10.1038/s41467-024-50467-x)
Supplement: Supplementary file 1 — Supplementary Information [file 41467_2024_50467_MOESM1_ESM.pdf]

Maternal Preconceptional and Prenatal Exposure to El Niño Southern Oscillation Levels and Child Mortality: A Multi-country Study

Hongbing XU, PhD [1,2]; Castiel Chen ZHUANG, PhD [3*]; Vanessa M. ODDO, PhD [4]; Espoir Bwenge MALEMBAKA, PhD [5, 6, 7]; Xinghou HE, MB [1,2]; Qinghong ZHANG, PhD [8]; Wei HUANG, ScD [1,2]

[1] Department of Occupational and Environmental Health, Peking University School of Public Health, Beijing, China;

[2] Peking University Institute of Environmental Medicine, Beijing, China;

[3] Peking University School of Economics, Beijing, China;

[4] Department of Kinesiology and Nutrition, College of Applied Health Sciences, University of Illinois Chicago, Chicago IL, USA;

[5] Center for Tropical Diseases and Global Health, Université Catholique de Bukavu, Bukavu, Democratic Republic of the Congo;

[6] Faculty of Medicine, Université Catholique de Bukavu, Bukavu, Democratic Republic of the Congo;

[7] Department of Epidemiology, Johns Hopkins Bloomberg School of Public Health, Baltimore MD, USA;

[8] Department of Atmospheric and Oceanic Sciences, School of Physics, Peking University, Beijing, China.

*Corresponding Author

Prof. Castiel Chen ZHUANG, PhD, Peking University School of Economics, No.5 Yiheyuan Road, Haidian District, Beijing 100871, China, Telephone: +86-010-62753423; Email: zogcee@gmail.com.

Supplementary Table S1. The raw sample size of each country in 38 LMICs.

Country, survey years	Geographic zone	Total births (n)	All-cause child deaths (n)		
			Neonatal (<1 month of age)	Infant (<12 months of age)	Under-five (<60 month of age)
Angola, 2015	Central Africa	14,134	306	549	682
Cameroon, 1991-2011	Central Africa	25,287	757	1,551	2,289
Chad, 1996-2014	Central Africa	31,488	1,037	2,308	3,445
Congo DR, 2007-2014	Central Africa	27,568	852	1,799	2,471
Zambia, 1992-2018	Central Africa	50,049	1,552	3,338	4,595
Burundi, 1987-2016	Eastern Africa	24,676	627	1,220	1,617
Kenya, 1989-2009	Eastern Africa	49,460	1,270	2,421	3,024
Madagascar, 1992-2008	Eastern Africa	26,571	752	1,496	1,925
Malawi, 1992-2016	Eastern Africa	64,298	2,039	4,277	5,757
Mozambique, 1997-2011	Eastern Africa	25,364	829	1,857	2,312
Rwanda, 1992-2014	Eastern Africa	44,348	1,406	2,836	3,767
Uganda, 1988-2016	Eastern Africa	49,369	1,400	2,997	4,061
Tanzania, 1991-2015	Eastern Africa	44,865	1,367	2,825	3,649
Morocco, 1987-2003	Northern Africa	17,462	574	948	1,102
Sudan, 1989-1990	Northern Africa	6,616	284	430	579
Tunisia, 1988	Northern Africa	4,477	117	203	227
Egypt, 1988-2014	Northern Africa	88,153	2,069	3,647	4,158
Lesotho, 2004-2014	Southern Africa	10,814	437	823	969
Namibia, 1992-2013	Southern Africa	18,052	429	791	1,011
South Africa, 1998-2016	Southern Africa	8,578	186	348	403
Zimbabwe, 1988-2015	Southern Africa	26,294	672	1,325	1,682
Benin, 1996-2018	Western Africa	51,274	1,504	2,871	3,915
Ghana, 1988-2014	Western Africa	22,330	785	1,252	1,660
Guinea, 1999-2018	Western Africa	26,842	1,032	1,995	2,766
Cote d'Ivoire, 1994-2011	Western Africa	13,652	524	963	1,264
Liberia, 1986-2013	Western Africa	16,515	538	1,048	1,366
Mali, 1987-2018	Western Africa	56,440	2,426	4,423	6,336
Niger, 1992-2012	Western Africa	33,243	983	2,223	3,729
Nigeria, 1990-2018	Western Africa	110,870	4,308	7,599	11,415
Senegal, 1986-2017	Western Africa	79,733	2,343	3,865	5,179
Burkina Faso, 1993-2010	Western Africa	37,392	1,177	2,687	4,167
Jordan, 1990-2018	Western Asia	62,000	942	1,367	1,511
Yemen, 1991-2013	Western Asia	23,301	704	1,205	1,405
Bangladesh, 1996-2014	South Asia	45,505	1,720	2,524	2,883
Sri Lanka, 1987	South Asia	4,006	62	92	105
India, 1992-2016	South Asia	392,281	12,645	18,147	20,361
Pakistan, 1990-2018	South Asia	39,946	1,697	2,509	2,792
Myanmar, 2015	Southeast Asia	4,812	126	197	218

Supplementary Table S2. The analyzed sample size of each country in 38 LMICs.

Country, survey years	Geographic zone	Total births (n)	All-cause child deaths (n)		
			Neonatal (<1 month of age)	Infant (<12 months of age)	Under-five (<60 month of age)
**Participants with data for all covariates**					
Angola, 2015	Central Africa	13,696	296	535	663
Cameroon, 1991-2011	Central Africa	24,746	698	1,464	2,161
Chad, 1996-2014	Central Africa	31,082	999	2,252	3,360
Congo DR, 2007-2014	Central Africa	27,049	801	1,695	2,338
Zambia, 1992-2018	Central Africa	42,973	1,235	2,610	3,585
Burundi, 1987-2016	Eastern Africa	19,818	473	905	1,161
Kenya, 1989-2009	Eastern Africa	41,054	973	1,843	2,278
Madagascar, 1992-2008	Eastern Africa	20,991	510	1,007	1,248
Malawi, 1992-2016	Eastern Africa	63,002	1,962	4,145	5,568
Mozambique, 1997-2011	Eastern Africa	24,445	753	1,729	2,168
Rwanda, 1992-2014	Eastern Africa	43,232	1,314	2,656	3,535
Uganda, 1988-2016	Eastern Africa	43,724	1,168	2,499	3,349
Tanzania, 1991-2015	Eastern Africa	36,132	1,047	2,077	2,693
Morocco, 1987-2003	Northern Africa	11,183	312	509	581
Egypt, 1988-2014	Northern Africa	69,389	1,423	2,445	2,732
Lesotho, 2004-2014	Southern Africa	10,455	412	788	931
Namibia, 1992-2013	Southern Africa	17,437	403	737	941
South Africa, 1998-2016	Southern Africa	8,327	167	310	354
Zimbabwe, 1992-2018	Southern Africa	22,429	561	1,127	1,430
Benin, 1996-2018	Western Africa	50,719	1,452	2,778	3,799
Ghana, 1988-2014	Western Africa	18,039	575	907	1,153
Guinea, 1999-2018	Western Africa	26,482	992	1,917	2,653
Cote d'Ivoire, 1994-2011	Western Africa	13,421	502	935	1,231
Liberia, 1986-2013	Western Africa	13,017	375	731	967
Mali, 1987-2018	Western Africa	42,704	1,902	3,536	5,057
Niger, 1992-2012	Western Africa	26,028	718	1,512	2,402
Nigeria, 1990-2018	Western Africa	105,061	3,967	7,007	10,579
Senegal, 1986-2017	Western Africa	68,626	1,912	3,081	3,990
Burkina Faso, 1993-2010	Western Africa	37,086	1,152	2,641	4,099
Jordan, 1990-2018	Western Asia	51,990	789	1,142	1,260
Bangladesh, 1996-2014	South Asia	42,195	1,605	2,364	2,701
India, 1992-2016	South Asia	388,996	12,259	17,581	19,725
Pakistan, 1990-2018	South Asia	39,410	1,620	2,390	2,652
Myanmar, 2015	Southeast Asia	4,789	123	193	213
**Participants without data for some covariates***					
Sudan, 1989-1990	Northern Africa	6,141	256	394	535
Tunisia, 1988	Northern Africa	4,361	110	193	217
Yemen, 1991-2013	Western Asia	22,190	492	842	958
Sri Lanka, 1987	South Asia	3,958	61	90	103

Notes: *Sri Lanka, Sudan, and Tunisia did not have covariates for child delivery location and household wealth; Yemen did not have covariates for educational level of mother and household wealth.

Supplementary Table S3. Characteristics of children under-five years of age and their mothers based on the raw sample.

Characteristics	Entire Cohort	All-cause child mortality		
		Neonatal (<1 month of age)	Infant (<12 months of age)	Under-five (<60 month of age)
Children, n	1,678,065	52,478	92,956	120,797
Total follow-up in month, n	44,704,084	0	202,220	779,735
Maternal covariates				
Mother age at child birth in years, mean (SD)	26.5 (6.4)	26.3 (7.1)	26.4 (7.0)	26.4 (7.0)
Mother age group in years, n (%)				
[15, 19]	254,540 (15.2)	10,874 (20.7)	18,632 (20.0)	23,599 (19.5)
[20, 24]	526,085 (31.4)	15,551 (29.6)	27,208 (29.3)	35,332 (29.2)
[25, 29]	432,658 (25.8)	11,188 (21.3)	20,658 (22.2)	27,236 (22.5)
[30, 34]	263,712 (15.7)	7,667 (14.6)	13,797 (14.8)	18,176 (15.0)
⩾35	201,070 (12.0)	7,198 (13.7)	12,661 (13.6)	16,454 (13.6)
Urbanicity, n (%)				
Urban	501,285 (29.9)	13,451 (25.6)	22,764 (24.5)	28,678 (23.7)
Rural	1,170,685 (69.8)	38,780 (73.9)	69,775 (75.1)	91,622 (75.8)
Missing	6,095 (0.4)	247 (0.5)	417 (0.4)	497 (0.4)
Marital status, n (%)				
In marriage	1,556,000 (92.7)	48,506 (92.4)	84,956 (91.4)	110,180 (91.2)
Others	122,035 (7.3)	3,972 (7.6)	7,999 (8.6)	10,616 (8.8)
Missing	30 (0.0)	0 (0.0)	1 (0.0)	1 (0.0)
Education, n (%)				
No education	695,227 (41.4)	25,188 (48.0)	46,205 (49.7)	62,698 (51.9)
Primary	465,240 (27.7)	14,749 (28.1)	27,654 (29.7)	36,014 (29.8)
Secondary or higher	501,273 (29.9)	12,127 (23.1)	18,445 (19.8)	21,363 (17.7)
Missing	16,325 (1.0)	414 (0.8)	652 (0.7)	722 (0.6)
Wealth quantile of household, n (%)				
Poorest or poorer—bottom 40%	711,048 (42.4)	23,984 (45.7)	42,818 (46.1)	56,154 (46.5)
Middle or richer—middle 40%	600,681 (35.8)	18,149 (34.6)	31,587 (34.0)	40,503 (33.5)
Richest—top 20%	250,999 (15.0)	6,124 (11.7)	10,245 (11.0)	12,665 (10.5)
Missing	115,337 (6.9)	4,221 (8.0)	8,306 (8.9)	11,475 (9.5)
Household access to safe water, n (%)				
Yes	631,674 (37.6)	16,389 (31.2)	27,636 (29.7)	34,166 (28.3)
No	1,024,924 (61.1)	35,408 (67.5)	64,134 (69.0)	85,102 (70.5)
Missing	21,467 (1.3)	681 (1.3)	1,186 (1.3)	1,529 (1.3)
Household access to toilet facilities, n (%)				
Yes	537,441 (32.0)	19,708 (37.6)	34,923 (37.6)	45,811 (37.9)
No	1,122,139 (66.9)	32,187 (61.3)	56,979 (61.3)	73,580 (60.9)
Missing	18,485 (1.1)	583 (1.1)	1,054 (1.1)	1,406 (1.2)
Child covariates				
Sex, n (%)				
Male	856,011 (51.0)	30,016 (57.2)	50,798 (54.6)	64,806 (53.6)
Female	822,054 (49.0)	22,462 (42.8)	42,158 (45.4)	55,991 (46.4)
Birth order, n (%)				
First	412,440 (24.6)	15,246 (29.1)	24,090 (25.9)	29,206 (24.2)
Second	360,376 (21.5)	9,529 (18.2)	16,880 (18.2)	21,764 (18.0)
Third or fourth	461,534 (27.5)	12,162 (23.2)	22,910 (24.6)	30,666 (25.4)
⩾Fifth	443,715 (26.4)	15,541 (29.6)	29,076 (31.3)	39,161 (32.4)
Child delivery place, n (%)				
Institution	695,708 (41.5)	23,500 (44.8)	44,557 (47.9)	60,184 (49.8)
Home	890,067 (53.0)	24,574 (46.8)	40,773 (43.9)	50,594 (41.9)
Missing	92,290 (5.5)	4,404 (8.4)	7,626 (8.2)	10,019 (8.3)
Geographic zone, n (%)				
Northern and Western Africa	571,851 (34.1)	17,896 (34.1)	26,041 (28.0)	29,275 (24.2)
Central, Eastern, and Southern Africa	541,215 (32.3)	15,918 (30.3)	32,761 (35.2)	43,659 (36.1)
South, Southeast and Western Asia	564,999 (33.7)	18,664 (35.6)	34,154 (36.7)	47,863 (39.6)

Notes: n, count or the number of observations; SD, standard deviation; %, percentage point. The numbers in the parentheses can be a SD or %, while the numbers outside the parentheses can be a mean or count, as denoted above.

Supplementary Table S4. Cumulative associations between child survival and extreme levels of maternal ENSO exposure at 0-12 months prior to delivery stratified by characteristics of study participants.

	Cooking fuels	
	Clean	Non-clean
**Neonatal mortality**		
MEI	1.20 (1.01, 1.43)	1.13 (1.06, 1.21)
P-value	Ref.	0.56
ESPI	1.33 (0.96, 1.84)	1.20 (1.06, 1.37)
P-value	Ref.	0.59
ONI	1.15 (0.91, 1.45)	1.11 (1.01, 1.22)
P-value	Ref.	0.77
Niño 1+2	1.40 (1.04, 1.88)	1.34 (1.20, 1.50)
P-value	Ref.	0.80
Niño 3.4	1.14 (0.91, 1.42)	1.1 (1.01, 1.20)
P-value	Ref.	0.78
**Infant mortality**		
MEI	1.21 (1.06, 1.40)	1.35 (1.28, 1.41)
P-value	Ref.	0.18
ESPI	1.37 (1.05, 1.79)	1.58 (1.44, 1.74)
P-value	Ref.	0.32
ONI	1.21 (1.00, 1.47)	1.33 (1.24, 1.42)
P-value	Ref.	0.39
Niño 1+2	1.31 (1.03, 1.65)	1.87 (1.73, 2.02)
P-value	Ref.	0.005
Niño 3.4	1.20 (0.99, 1.44)	1.30 (1.21, 1.39)
P-value	Ref.	0.41
**Under-five Mortality**		
MEI	1.24 (1.08, 1.42)	1.49 (1.42, 1.55)
P-value	Ref.	0.01
ESPI	1.37 (1.06, 1.77)	1.90 (1.75, 2.07)
P-value	Ref.	0.02
ONI	1.22 (1.01, 1.47)	1.46 (1.37, 1.55)
P-value	Ref.	0.08
Niño 1+2	1.33 (1.06, 1.66)	2.33 (2.18, 2.50)
P-value	Ref.	<0.001
Niño 3.4	1.20 (1.00, 1.44)	1.41 (1.33, 1.50)
P-value	Ref.	0.09

Notes: *P*-values are for the differences in effect estimates across subgroups. The effect estimate of each ENSO measure with child survival is computed as the hazard ratio of a given percentile of ENSO measure relative to the reference value (set at zero). Abbreviations: ENSO, El Niño Southern Oscillation; MEI, multivariate El Niño index; ESPI, ENSO precipitation index; ONI, oceanic Niño index; Ref., reference group. The differential association estimates between a subgroup and the reference are tested using the two-sided two-sample z-test given by Equation (1) presented in the main text. The z-statistic in the last but one row is 4.74, and the exact *P*-value is 2.10×10^{-6}. Adjustments are not made for multiple comparisons.

Supplementary Table S5. Cumulative associations between child survival and extreme levels of ENSO exposure at 0-12 preconceptional and prenatal months of mothers stratified by levels of meteorological parameters at each DHS cluster location.

	Temperature			Precipitation		
	first tertile	second tertile	third tertile	first tertile	second tertile	third tertile
**Neonatal mortality**						
MEI	1.20 (0.96, 1.50)	1.24 (1.00, 1.52)	1.23 (1.02, 1.49)	1.21 (1.03, 1.43)	1.07 (0.91, 1.25)	1.02 (0.87, 1.21)
P-value	Ref.	0.47	0.35	Ref.	0.91	0.13
ESPI	1.31 (1.00, 1.72)	1.40 (1.08, 1.81)	1.18 (0.94, 1.48)	1.15 (0.91, 1.46)	1.31 (1.04, 1.65)	1.23 (0.97, 1.57)
P-value	Ref.	0.73	0.56	Ref.	0.45	0.69
ONI	1.29 (1.12, 1.49)	1.20 (1.04, 1.38)	1.18 (1.05, 1.33)	1.20 (1.07, 1.36)	1.22 (1.08, 1.38)	1.05 (0.92, 1.19)
P-value	Ref.	0.86	0.87	Ref.	0.28	0.16
Niño 1+2	1.47 (1.16, 1.85)	1.69 (1.33, 2.14)	1.27 (1.05, 1.53)	1.22 (0.99, 1.51)	1.51 (1.23, 1.87)	1.57 (1.26, 1.96)
P-value	Ref.	0.41	0.35	Ref.	0.16	0.11
Niño 3.4	1.18 (0.95, 1.47)	1.17 (0.96, 1.44)	1.18 (0.98, 1.43)	1.19 (1.02, 1.4)	1.03 (0.89, 1.20)	1.01 (0.86, 1.19)
P-value	Ref.	0.95	0.99	Ref.	0.21	0.15
**Infant mortality**						
MEI	1.51 (1.36, 1.67)	1.49 (1.35, 1.66)	1.36 (1.25, 1.48)	1.34 (1.23, 1.47)	1.44 (1.32, 1.57)	1.24 (1.13, 1.37)
P-value	Ref.	0.90	0.12	Ref.	0.28	0.26
ESPI	1.66 (1.36, 2.02)	1.85 (1.53, 2.25)	1.47 (1.25, 1.73)	1.25 (1.05, 1.49)	1.78 (1.50, 2.10)	1.51 (1.26, 1.81)
P-value	Ref.	0.43	0.35	Ref.	0.004	0.13
ONI	1.52 (1.29, 1.79)	1.61 (1.38, 1.89)	1.43 (1.24, 1.64)	1.32 (1.17, 1.51)	1.33 (1.18, 1.49)	1.18 (1.04, 1.34)
P-value	Ref.	0.13	0.55	Ref.	0.99	0.21
Niño 1+2	1.87 (1.59, 2.20)	2.22 (1.88, 2.62)	1.61 (1.41, 1.84)	1.28 (1.10, 1.48)	2.22 (1.93, 2.56)	2.06 (1.76, 2.41)
P-value	Ref.	0.15	0.16	Ref.	<0.001	<0.001
Niño 3.4	1.47 (1.25, 1.73)	1.52 (1.30, 1.78)	1.37 (1.19, 1.57)	1.30 (1.15, 1.47)	1.28 (1.14, 1.43)	1.15 (1.02, 1.30)
P-value	Ref.	0.78	0.50	Ref.	0.86	0.19
**Under-five Mortality**						
MEI	1.60 (1.46, 1.75)	1.56 (1.42, 1.71)	1.45 (1.36, 1.56)	1.41 (1.31, 1.53)	1.53 (1.42, 1.64)	1.32 (1.21, 1.44)
P-value	Ref.	0.67	0.10	Ref.	0.16	0.25
ESPI	1.86 (1.56, 2.22)	1.94 (1.63, 2.32)	1.55 (1.35, 1.78)	1.26 (1.08, 1.47)	1.85 (1.60, 2.14)	1.63 (1.38, 1.91)
P-value	Ref.	0.75	0.11	Ref.	<0.001	0.02
ONI	1.65 (1.42, 1.91)	1.69 (1.46, 1.95)	1.50 (1.34, 1.69)	1.33 (1.19, 1.49)	1.37 (1.23, 1.51)	1.22 (1.08, 1.36)
P-value	Ref.	0.82	0.33	Ref.	0.76	0.26
Niño 1+2	2.18 (1.90, 2.51)	2.25 (1.94, 2.61)	1.81 (1.63, 2.02)	1.34 (1.18, 1.53)	2.57 (2.28, 2.89)	2.23 (1.95, 2.57)
P-value	Ref.	0.78	0.04	Ref.	<0.001	<0.001
Niño 3.4	1.59 (1.38, 1.84)	1.58 (1.36, 1.82)	1.42 (1.27, 1.60)	1.30 (1.16, 1.45)	1.31 (1.19, 1.45)	1.18 (1.05, 1.32)
P-value	Ref.	0.93	0.23	Ref.	0.89	0.22

Notes: *P*-values are for the differences in effect estimates across subgroups. The effect estimate of each ENSO measure with child survival is computed as the hazard ratio of a given percentile of ENSO relative to the reference value (set at zero). Abbreviations: ENSO, El Niño Southern Oscillation; MEI, multivariate El Niño index; ESPI, ENSO precipitation index; ONI, oceanic Niño index; Ref., reference group. The differential association estimates between a subgroup and the reference are tested using the two-sided two-sample z-test given by Equation (1) presented in the main text. The z-statistics for precipitation's second and third tertiles based on Niño 1+2 are 5.26 and 4.28 for infant mortality, and the corresponding exact *P*-values are 1.44×10^{-7} and 1.84×10^{-5}; for under-five mortality, the z-statistics for precipitation's second and third tertiles based on Niño 1+2 are 7.25 and 5.28, and the exact *P*-values are 4.30×10^{-13} and 1.30×10^{-7}; for under-five mortality, the z-statistics for precipitation's second tertile based on ESPI is 3.59, and the exact *P*-value is 3.26×10^{-4}. Adjustments are not made for multiple comparisons.

Supplementary Table S6. Attributable burden of child mortality related to extreme levels of ENSO exposure measured by MEI, ESPI, and ONI at 0-12 preconceptional and prenatal months of mothers.

		Total births (n)	Death numbers (n)	HR (95%CI)	AF (%) (95% CI)	Attributable deaths (n) (95% CI)
**MEI**						
Neonatal mortality	La Niña (≤ -0.5)	494,756	14,755	0.99 (0.97, 1.01)	-1.2 (-3.4, 0.8)	NA
	Neutral (-0.5 to +0.5)	805,824	24,066	Ref.	Ref.	--
	Weak El Niño (≥ +0.5 to < +1.0)	95,823	3,152	1.10 (1.06, 1.14)	8.7 (5.2, 12.1)	275 (165, 381)
	Moderate El Niño (≥ +1.0)	103,324	3,477	1.12 (1.08, 1.16)	10.4 (7.1, 13.5)	362 (248, 471)
Infant mortality	La Niña (≤ -0.5)	494,756	26,076	0.98 (0.96, 0.99)	-2.2 (-3.9, -0.7)	NA
	Neutral (-0.5 to +0.5)	805,824	41,744	Ref.	Ref.	--
	Weak El Niño (≥ +0.5 to < +1.0)	95,823	6,074	1.19 (1.16, 1.22)	15.9 (13.5, 18.1)	964 (823, 1,102)
	Moderate El Niño (≥ +1.0)	103,324	6,154	1.21 (1.17, 1.24)	17.0 (14.7, 19.2)	1,047 (907, 1,183)
Under-five mortality	La Niña (≤ -0.5)	494,756	34,393	0.96 (0.95, 0.97)	-4.2 (-5.6, -2.8)	NA
	Neutral (-0.5 to +0.5)	805,824	53,337	Ref.	Ref.	--
	Weak El Niño (≥ +0.5 to < +1.0)	95,823	7,944	1.22 (1.20, 1.25)	18.4 (16.4, 20.3)	1,458 (1,301, 1,612)
	Moderate El Niño (≥ +1.0)	103,324	7,883	1.26 (1.23, 1.29)	20.9 (19.0, 22.8)	1,647 (1,496, 1,794)
**ESPI**						
Neonatal mortality	La Niña (≤ -0.5)	529,839	15,846	0.99 (0.98, 1.02)	-0.2 (-2.3, 1.9)	NA
	Neutral (-0.5 to +0.5)	697,174	20,676	Ref.	Ref.	--
	Weak El Niño (≥ +0.5 to < +1.0)	159,361	5,240	1.10 (1.06, 1.13)	8.9 (6.1, 11.6)	465 (317, 609)
	Moderate El Niño (≥ +1.0)	113,353	3,688	1.09 (1.05, 1.13)	8.4 (5.1, 11.5)	308 (187, 425)
Infant mortality	La Niña (≤ -0.5)	529,839	27,862	0.99 (0.97, 1.00)	-1.4 (-3.0, 0.2)	NA
	Neutral (-0.5 to +0.5)	697,174	36,008	Ref.	Ref.	--
	Weak El Niño (≥ +0.5 to < +1.0)	159,361	9,724	1.14 (1.11, 1.17)	12.2 (10.2, 14.2)	1,190 (995, 1,380)
	Moderate El Niño (≥ +1.0)	113,353	6,454	1.17 (1.14, 1.20)	14.7 (12.4, 17.0)	951 (802, 1,096)
Under-five mortality	La Niña (≤ -0.5)	529,839	36,622	0.97 (0.95, 0.98)	-3.4 (-4.8, -2.0)	NA
	Neutral (-0.5 to +0.5)	697,174	46,260	Ref.	Ref.	--
	Weak El Niño (≥ +0.5 to < +1.0)	159,361	12,568	1.14 (1.12, 1.16)	12.4 (10.6, 14.1)	1,556 (1,335, 1,773)
	Moderate El Niño (≥ +1.0)	113,353	8,107	1.22 (1.19, 1.25)	18.2 (16.2, 20.1)	1,476 (1,317, 1,632)
**ONI**						
Neonatal mortality	La Niña (≤ -0.5)	394,180	11,825	0.99 (0.97, 1.01)	-0.8 (-3.1, 1.3)	NA
	Neutral (-0.5 to +0.5)	830,008	24,898	Ref.	Ref.	--
	Weak El Niño (≥ +0.5 to < +1.0)	189,117	5,939	1.04 (1.01, 1.07)	3.7 (0.9, 6.4)	218 (53, 378)
	Moderate El Niño (≥ +1.0)	86,422	2,788	1.07 (1.03, 1.11)	6.7 (3.0, 10.3)	187 (83, 287)
Infant mortality	La Niña (≤ -0.5)	394,180	21,027	0.98 (0.97, 0.99)	-1.8 (-3.5, -0.2)	NA
	Neutral (-0.5 to +0.5)	830,008	43,397	Ref.	Ref.	--
	Weak El Niño (≥ +0.5 to < +1.0)	189,117	10,746	1.05 (1.03, 1.08)	5.1 (3.0, 7.1)	545 (326, 758)
	Moderate El Niño (≥ +1.0)	86,422	4,878	1.15 (1.12, 1.18)	13.0 (10.4, 15.6)	636 (508, 760)
Under-five mortality	La Niña (≤ -0.5)	394,180	27,804	0.96 (0.95, 0.97)	-4.2 (-5.8, -2.7)	NA
	Neutral (-0.5 to +0.5)	830,008	55,787	Ref.	Ref.	--
	Weak El Niño (≥ +0.5 to < +1.0)	189,117	13,799	1.05 (1.03, 1.07)	4.5 (2.7, 6.3)	626 (377, 870)
	Moderate El Niño (≥ +1.0)	86,422	6,167	1.19 (1.16, 1.22)	16.1 (13.8, 18.3)	991 (852, 1,126)

Notes: NA indicates no calculation results of attributable deaths when there was a negative value of AF. Abbreviations: HR, hazard ratio; AF, attributable fraction; CI, confidence interval; ENSO, El Niño Southern Oscillation; MEI, multivariate El Niño index; ESPI, ENSO precipitation index; ONI, oceanic Niño index; Ref., reference group.

Supplementary Table S7. Attributable burden of child mortality related to extreme levels of ENSO exposure measured by Niño 1+2 and Niño 3.4 at 0-12 preconceptional and prenatal months of mothers.

	Total births (n)	Death numbers (n)	HR (95%CI)	AF (%) (95% CI)	Attributable deaths (n) (95% CI)
**Niño 1+2**					
Neonatal mortality					
La Niña (≤ -0.5)	452,671	13,628	1.01 (0.99, 1.03)	0.7 (-1.4, 2.7)	89 (-197, 368)
Neutral (-0.5 to +0.5)	845,417	25,253	Ref.	Ref.	-
Weak El Niño (≥ +0.5 to < +1.0)	93,266	3,047	1.09 (1.05, 1.13)	7.9 (4.4, 11.3)	241 (133, 345)
Moderate El Niño (≥ +1.0)	108,373	3,522	1.09 (1.05, 1.13)	8.0 (4.7, 11.2)	282 (165, 395)
Infant mortality					
La Niña (≤ -0.5)	452,671	24,154	1.01 (0.99, 1.03)	1.4 (-0.1, 3.0)	349 (-28, 721)
Neutral (-0.5 to +0.5)	845,417	44,369	Ref.	Ref.	-
Weak El Niño (≥ +0.5 to < +1.0)	93,266	5,387	1.16 (1.12, 1.19)	13.5 (11.0, 16.0)	729 (594, 859)
Moderate El Niño (≥ +1.0)	108,373	6,138	1.17 (1.14, 1.20)	14.3 (12.0, 16.6)	879 (736, 1,019)
Under-five mortality					
La Niña (≤ -0.5)	452,671	31,764	1.01 (0.99, 1.03)	0.7 (-1.4, 2.7)	207 (-459, 859)
Neutral (-0.5 to +0.5)	845,417	57,266	Ref.	Ref.	-
Weak El Niño (≥ +0.5 to < +1.0)	93,266	6,824	1.09 (1.05, 1.13)	7.9 (4.4, 11.3)	540 (297, 773)
Moderate El Niño (≥ +1.0)	108,373	7,703	1.09 (1.05, 1.13)	8.0 (4.7, 11.2)	617 (361, 864)
**Niño 3.4**					
Neonatal mortality					
La Niña (≤ -0.5)	415,685	12,522	1.00 (0.98, 1.02)	-0.2 (-2.4, 1.9)	NA
Neutral (-0.5 to +0.5)	832,074	24,903	Ref.	Ref.	--
Weak El Niño (≥ +0.5 to < +1.0)	173,817	5,529	1.06 (1.02, 1.09)	5.2 (2.4, 8.0)	290 (134, 441)
Moderate El Niño (≥ +1.0)	78,151	2,496	1.07 (1.02, 1.11)	6.2 (2.2, 10.0)	155 (56, 249)
Infant mortality					
La Niña (≤ -0.5)	415,685	22,324	0.99 (0.97, 1.00)	-1.2 (-2.9, 0.4)	NA
Neutral (-0.5 to +0.5)	832,074	43,417	Ref.	Ref.	--
Weak El Niño (≥ +0.5 to < +1.0)	173,817	9,992	1.07 (1.05, 1.09)	6.5 (4.4, 8.5)	646 (440, 848)
Moderate El Niño (≥ +1.0)	78,151	4,315	1.14 (1.11, 1.18)	12.4 (9.6, 15.1)	536 (415, 653)
Under-five mortality					
La Niña (≤ -0.5)	415,685	29,634	0.97 (0.96, 0.98)	-3.1 (-4.6, -1.7)	NA
Neutral (-0.5 to +0.5)	832,074	55,638	Ref.	Ref.	--
Weak El Niño (≥ +0.5 to < +1.0)	173,817	12,890	1.07 (1.05, 1.09)	6.4 (4.6, 8.2)	829 (594, 1,058)
Moderate El Niño (≥ +1.0)	78,151	5,395	1.18 (1.15, 1.22)	15.3 (12.9, 17.7)	828 (698, 955)

Notes: NA indicates no calculation results of attributable deaths when there was a negative value of AF. Abbreviations: HR, hazard ratio; AF, attributable fraction; CI, confidence interval; ENSO, El Niño Southern Oscillation; Ref., reference group.

Supplementary Table S8. Detailed information on the time series of each ENSO indicator.

ENSO indicator	Definition
MEI	Monthly indicator that incorporates sea-surface temperature (SST), winds, sea-level pressure (SLP), and outgoing long-wave radiation into a single multivariable index.
ESPI	It is based on rainfall anomalies in two rectangular areas, one in the eastern tropical Pacific (10°S-10°N, 160°E-100°W) and the other over the Maritime Continent (10°S-10°N, 90°E-150°E).
ONI	Three month running mean of ERSST.v4 SST anomalies in the Niño 3.4 region, based on changing base period which consists of multiple centered 30-year base periods. It is calculated from the Monthly NOAA ERSST V5 (at NOAA/CPC).
Niño 1+2	SST anomalies (1981-2010 mean removed) averaged over the Niño 1+2 region, the smallest and eastern-most of the Niño SST regions (0-10°S, 90°W-80°W). It is calculated from HadISST1.
Niño 3.4	SST anomalies (1981-2010 mean removed) averaged over the Niño 3.4 region (5°N-5°S, 170°W-120°W). It is calculated from HadISST1.

Abbreviations: ENSO, El Niño-Southern Oscillation; MEI, Multivariate El Niño Index; ESPI, ENSO Precipitation Index; ONI, Oceanic Niño Index; NOAA, National Oceanic and Atmospheric Administration; CPC, Climate Prediction Center; ERSST, Extended Reconstructed Sea Surface Temperature; HadISST1, Hadley Centre Global Sea Ice and Sea Surface Temperature data set.

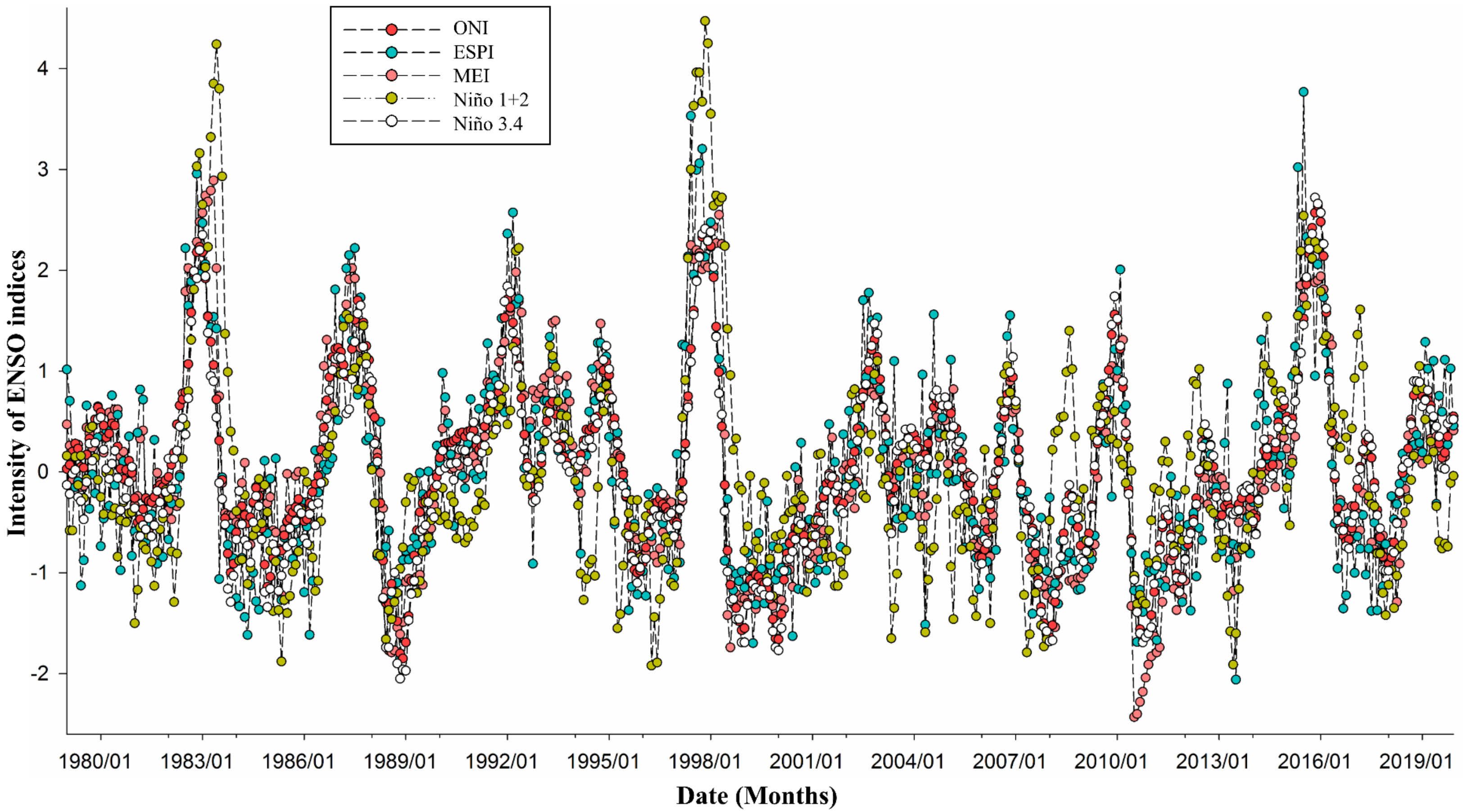

Supplementary Figure S1. The time-series pattern of ENSO indices throughout the study period. Abbreviations: ENSO, El Niño Southern Oscillation; MEI, multivariate El Niño index; ESPI, ENSO precipitation index; ONI, oceanic Niño index.

Characteristics	Subgroup	Neonatal mortality HR (95%CI)	Effect modification
Sex			
	Male	1.18(1.10,1.27)	Ref.
	Female	1.18(1.08,1.29)	0.99
Birth order			
	First	1.15(1.04,1.27)	Ref.
	Second	1.21(1.07,1.38)	0.52
	Third or fourth	1.12(0.99,1.26)	0.76
	≥Fifth	1.19(1.08,1.32)	0.61
Mother age			
	[15, 19)	1.20(1.06,1.34)	Ref.
	[20, 24)	1.15(1.03,1.27)	0.60
	[25, 29)	1.13(1.00,1.28)	0.53
	[30, 34)	1.16(1.00,1.35)	0.78
	≥35	1.25(1.07,1.46)	0.65
Urbanicity			
	Urban	1.14(1.02,1.28)	Ref.
	Rural	1.19(1.11,1.27)	0.53
Child delivery place			
	Institution	1.22(1.12,1.32)	Ref.
	Home	1.13(1.04,1.22)	0.18
Birth quarter	Q1	1.14(1.01,1.29)	Ref.
	Q2	1.11(0.99,1.24)	0.79
	Q3	1.29(1.12,1.48)	0.19
	Q4	1.29(1.13,1.48)	0.18
Safe water			
	Yes	1.23(1.11,1.35)	Ref.
	No	1.15(1.08,1.23)	0.33
Education			
	No education	1.16(1.07,1.25)	Ref.
	Primary	1.16(1.04,1.29)	0.97
	Secondary or higher	1.15(1.02,1.30)	0.99
Household wealth			
	Poorest or poorer	1.12(1.03,1.21)	Ref.
	Middle or richer	1.28(1.17,1.39)	0.03
	Richest	1.17(0.99,1.36)	0.65
Geographic zone			
	South, Southeast and Western Asia	1.07(0.98,1.18)	Ref.
	Northern and Western Africa	1.10(1.00,1.21)	0.73
	Central, Eastern and Southern Africa	1.42(1.28,1.58)	<0.001

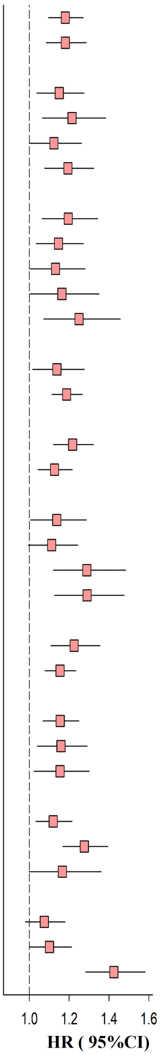

Supplementary Figure S2. Cumulative associations between neonatal mortality and high level of MEI (90th percentile) at 0-12 preconceptional and prenatal months of mothers stratified by characteristics of study participants. Notes: The sample sizes used to derive statistics are provided in Table 1. The effect estimate of MEI on child survival computed as the HR of the 90th percentile of MEI relative to the reference value (defined at 0) is presented as the center of error bars, and the error bars are represent 95% confidence intervals of the estimates. Abbreviations: HR, hazard ratio; CI, confidence interval; MEI, multivariate El Niño index. The differential association estimates between a subgroup and the reference (Ref.) are tested using the two-sided two-sample z-test given by Equation (1) presented in the main text. Adjustments are not made for multiple comparisons.

Characteristics	Subgroup	Infant mortality HR (95%CI)	Infant mortality Effect modification	Under-five mortality HR (95%CI)	Under-five mortality Effect modification	Neonatal mortality HR (95%CI)	Neonatal mortality Effect modification
Sex							
	Male	1.59(1.45,1.74)	Ref.	1.81(1.67,1.96)	Ref.	1.37(1.22,1.55)	Ref.
	Female	1.76(1.60,1.94)	0.12	1.98(1.82,2.15)	0.15	1.36(1.18,1.56)	0.92
Birth order							
	First	1.66(1.47,1.89)	Ref.	1.87(1.67,2.10)	Ref.	1.39(1.18,1.64)	Ref.
	Second	1.67(1.43,1.94)	0.99	1.93(1.68,2.21)	0.76	1.39(1.12,1.71)	0.99
	Third or fourth	1.60(1.40,1.84)	0.70	1.86(1.66,2.10)	0.95	1.21(1.00,1.47)	0.29
	≥Fifth	1.64(1.46,1.85)	0.88	1.81(1.63,2.01)	0.65	1.36(1.15,1.60)	0.85
Mother age							
	[15, 19)	1.66(1.45,1.90)	Ref.	1.88(1.66,2.13)	Ref.	1.43(1.19,1.71)	Ref.
	[20, 24)	1.67(1.48,1.89)	0.93	1.96(1.76,2.18)	0.61	1.29(1.09,1.53)	0.43
	[25, 29)	1.61(1.39,1.86)	0.76	1.80(1.59,2.04)	0.64	1.26(1.02,1.54)	0.36
	[30, 34)	1.53(1.28,1.83)	0.48	1.70(1.45,1.99)	0.32	1.24(0.97,1.60)	0.38
	≥35	1.75(1.45,2.11)	0.66	1.94(1.65,2.28)	0.76	1.58(1.22,2.03)	0.53
Urbanicity							
	Urban	1.33(1.15,1.53)	Ref.	1.53(1.34,1.74)	Ref.	1.24(1.02,1.51)	Ref.
	Rural	1.78(1.65,1.91)	<0.001	2.00(1.88,2.14)	<0.001	1.40(1.26,1.55)	0.28
Child delivery place							
	Institution	1.72(1.54,1.91)	Ref.	2.01(1.83,2.2)	Ref.	1.47(1.28,1.70)	Ref.
	Home	1.57(1.45,1.71)	0.21	1.78(1.66,1.92)	0.05	1.22(1.08,1.38)	0.05
Birth quarter	Q1	2.38(1.79,3.16)	Ref.	3.24(2.52,4.15)	Ref.	1.25(0.85,1.86)	Ref.
	Q2	4.44(3.15,6.25)	0.006	6.49(4.81,8.77)	<0.001	2.64(1.67,4.19)	0.02
	Q3	2.19(1.81,2.65)	0.63	2.74(2.32,3.24)	0.27	1.56(1.2,2.020)	0.36
	Q4	2.60(2.13,3.16)	0.62	3.32(2.80,3.95)	0.86	1.87(1.42,2.45)	0.10
Safe water							
	Yes	1.41(1.25,1.60)	Ref.	1.65(1.47,1.84)	Ref.	1.34(1.14,1.59)	Ref.
	No	1.78(1.64,1.92)	0.002	1.99(1.86,2.13)	0.005	1.37(1.23,1.52)	
Education							
	No education	1.67(1.52,1.82)	Ref.	1.87(1.72,2.02)	Ref.	1.37(1.21,1.55)	Ref.
	Primary	1.83(1.62,2.07)	0.21	2.16(1.95,2.40)	0.03	1.28(1.07,1.53)	0.55
	Secondary or higher	1.28(1.09,1.51)	0.007	1.40(1.20,1.64)		1.19(0.97,1.46)	0.26
Household wealth							
	Poorest or poorer	1.75(1.59,1.93)	Ref.	1.96(1.80,2.13)	Ref.	1.34(1.17,1.53)	Ref.
	Middle or richer	1.71(1.54,1.90)	0.72	1.96(1.79,2.15)	0.95	1.46(1.26,1.68)	0.38
	Richest	1.46(1.21,1.76)	0.09	1.68(1.42,1.99)	0.12	1.33(1.04,1.72)	0.99
Geographic zone							
	South, Southeast and Western Asia	1.13(1.00,1.28)	Ref.	1.11(0.99,1.25)	Ref.	1.14(0.99,1.33)	Ref.
	Northern and Western Africa	1.65(1.47,1.85)	<0.001	1.82(1.65,2.00)	<0.001	1.33(1.13,1.56)	0.18
	Central, Eastern and Southern Africa	2.25(2.01,2.51)	<0.001	2.47(2.24,2.71)	<0.001	1.67(1.41,1.97)	<0.001

Forest plot axes: Infant mortality: 1 2 3 4 5 6, HR (95%CI); Under-five mortality: 1 2 3 4 5 6 7 8, HR (95%CI); Neonatal mortality: 1 2 3 4, HR (95%CI)

Supplementary Figure S3. Cumulative associations between child mortality and high level of Niño 1+2 (90th percentile) at 0-12 preconceptional and prenatal months of mothers stratified by characteristics of study participants. Notes: The sample sizes used to derive statistics are provided in Table 1. The effect estimate of Niño 1+2 on child survival computed as the HR of the 90th percentile of Niño 1+2 relative to the reference value (defined at 0) is presented as the center of error bars, and the error bars are represent 95% confidence intervals of the estimates. Abbreviations: HR, hazard ratio; CI, confidence interval. The differential association estimates between a subgroup and the reference (Ref.) are tested using the two-sided two-sample z-test given by Equation (1) presented in the main text. Adjustments are not made for multiple comparisons.

		Infant mortality		Under-five mortality		Neonatal mortality	
Characteristics	Subgroup	HR (95%CI)	Effect modification	HR (95%CI)	Effect modification	HR (95%CI)	Effect modification
Sex							
	Male	1.23(1.13,1.33)	Ref.	1.31(1.22,1.41)	Ref.	1.12(1.01,1.24)	Ref.
	Female	1.32(1.21,1.44)	0.21	1.36(1.26,1.47)	0.46	1.12(1.00,1.26)	0.99
Birth order							
	First	1.18(1.05,1.33)	Ref.	1.29(1.16,1.43)	Ref.	0.99(0.86,1.15)	Ref.
	Second	1.34(1.17,1.53)	0.17	1.43(1.27,1.62)	0.20	1.22(1.02,1.45)	0.09
	Third or fourth	1.13(1.01,1.28)	0.64	1.22(1.10,1.35)	0.48	1.09(0.93,1.28)	0.41
	≥Fifth	1.38(1.25,1.53)	0.05	1.36(1.24,1.49)	0.46	1.20(1.05,1.39)	0.07
Mother age							
	[15, 19)	1.29(1.13,1.48)	Ref.	1.41(1.25,1.59)	Ref.	1.09(0.92,1.30)	Ref.
	[20, 24)	1.26(1.13,1.41)	0.79	1.38(1.25,1.52)	0.75	1.11(0.96,1.28)	0.89
	[25, 29)	1.09(0.96,1.24)	0.06	1.19(1.06,1.33)	0.04	1.05(0.89,1.24)	0.75
	[30, 34)	1.36(1.17,1.58)	0.64	1.26(1.10,1.45)	0.22	1.24(1.02,1.50)	0.34
	≥35	1.43(1.22,1.67)	0.35	1.43(1.25,1.64)	0.89	1.18(0.96,1.44)	0.57
Urbanicity							
	Urban	1.10(0.98,1.24)	Ref.	1.17(1.05,1.31)	Ref.	1.08(0.93,1.26)	Ref.
	Rural	1.33(1.25,1.43)	0.006	1.39(1.31,1.48)	0.007	1.14(1.04,1.24)	0.57
Child delivery place							
	Institution	1.12(1.03,1.22)	Ref.	1.17(1.08,1.27)	Ref.	1.09(0.98,1.21)	Ref.
	Home	1.39(1.28,1.51)	<0.001	1.45(1.35,1.56)	<0.001	1.11(0.99,1.24)	0.83
Birth quarter	Q1	1.78(1.35,2.34)	Ref.	2.13(1.68,2.71)	Ref.	1.37(0.95,1.99)	Ref.
	Q2	1.44(0.90,2.33)	0.46	2.13(1.41,3.24)	0.99	0.75(0.39,1.44)	0.12
	Q3	1.62(1.12,2.36)	0.70	2.14(1.54,2.97)	0.99	1.40(0.85,2.30)	0.96
	Q4	0.92(0.72,1.17)	<0.001	0.92(0.74,1.14)	<0.001	1.04(0.75,1.45)	0.28
Safe water							
	Yes	1.18(1.06,1.31)	Ref.	1.24(1.12,1.37)	Ref.	1.15(1.00,1.32)	Ref.
	No	1.31(1.22,1.40)	0.12	1.37(1.28,1.45)	0.11	1.11(1.01,1.21)	0.65
Education							
	No education	1.29(1.19,1.40)	Ref.	1.34(1.24,1.44)	Ref.	1.12(1.01,1.25)	Ref.
	Primary	1.32(1.18,1.48)	0.75	1.46(1.32,1.61)	0.17	1.08(0.93,1.26)	0.70
	Secondary or higher	1.10(0.97,1.25)	0.04	1.12(1.00,1.27)	0.01	1.12(0.96,1.30)	0.98
Household wealth							
	Poorest or poorer	1.27(1.17,1.38)	Ref.	1.31(1.22,1.41)	Ref.	1.06(0.95,1.19)	Ref.
	Middle or richer	1.31(1.19,1.44)	0.61	1.41(1.29,1.53)	0.20	1.22(1.08,1.38)	0.11
	Richest	1.17(0.98,1.39)	0.41	1.23(1.05,1.45)	0.52	1.09(0.87,1.35)	0.87
Geographic zone							
	South, Southeast and Western Asia	1.17(1.05,1.30)	Ref.	1.15(1.04,1.28)	Ref.	1.09(0.96,1.24)	Ref.
	Northern and Western Africa	1.11(1.01,1.22)	0.47	1.08(1.00,1.17)	0.35	1.02(0.90,1.15)	0.46
	Central, Eastern and Southern Africa	1.55(1.39,1.73)	<0.001	1.68(1.53,1.86)	<0.001	1.36(1.16,1.59)	0.03

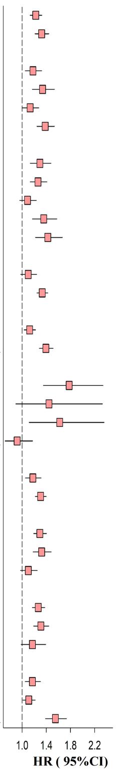

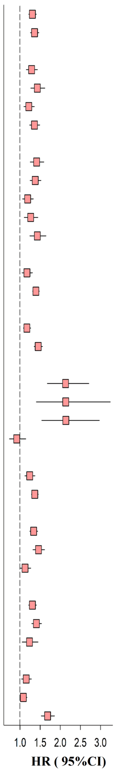

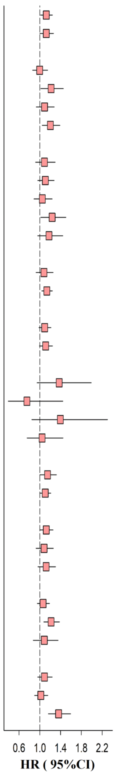

Supplementary Figure S4. Cumulative associations between child mortality and high level of Niño 3.4 (90th percentile) at 0-12 preconceptional and prenatal months of mothers stratified by characteristics of study participants. Notes: The sample sizes used to derive statistics are provided in Table 1. The effect estimate of Niño 1+2 on child survival computed as the HR of the 90th percentile of Niño 1+2 relative to the reference value (defined at 0) is presented as the center of error bars, and the error bars are represent 95% confidence intervals of the estimates. Abbreviations: HR, hazard ratio; CI, confidence interval. The differential association estimates between a subgroup and the reference (Ref.) are tested using the two-sided two-sample z-test given by Equation (1) presented in the main text. Adjustments are not made for multiple comparisons.

Characteristics	Subgroup	Infant mortality HR (95%CI)	Infant mortality Effect modification	Under-five mortality HR (95%CI)	Under-five mortality Effect modification	Neonatal mortality HR (95%CI)	Neonatal mortality Effect modification
Sex							
	Male	1.43(1.29,1.60)	Ref.	1.60(1.45,1.76)	Ref.	1.29(1.12,1.49)	Ref.
	Female	1.43(1.27,1.61)	0.96	1.54(1.39,1.71)	0.62	1.14(0.97,1.35)	0.27
Birth order							
	First	1.40(1.20,1.64)	Ref.	1.55(1.35,1.79)	Ref.	1.16(0.95,1.42)	Ref.
	Second	1.46(1.21,1.76)	0.75	1.64(1.39,1.94)	0.62	1.33(1.03,1.71)	0.41
	Third or fourth	1.22(1.03,1.44)	0.22	1.39(1.20,1.61)	0.29	1.06(0.85,1.34)	0.57
	≥Fifth	1.56(1.35,1.81)	0.32	1.61(1.42,1.83)	0.70	1.32(1.08,1.62)	0.37
Mother age							
	[15, 19)	1.56(1.31,1.85)	Ref.	1.71(1.46,2.00)	Ref.	1.38(1.1,1.74)	Ref.
	[20, 24)	1.36(1.17,1.58)	0.25	1.60(1.40,1.83)	0.52	1.11(0.91,1.36)	0.16
	[25, 29)	1.24(1.04,1.47)	0.06	1.38(1.18,1.61)	0.06	1.07(0.85,1.36)	0.13
	[30, 34)	1.33(1.07,1.65)	0.26	1.37(1.13,1.66)	0.07	1.15(0.86,1.53)	0.32
	≥35	1.80(1.44,2.25)	0.32	1.82(1.50,2.22)	0.62	1.51(1.12,2.04)	0.65
Urbanicity							
	Urban	1.10(0.93,1.31)	Ref.	1.22(1.05,1.43)	Ref.	1.07(0.86,1.34)	Ref.
	Rural	1.55(1.41,1.69)	<0.001	1.69(1.56,1.83)	<0.001	1.27(1.12,1.44)	0.20
Child delivery place							
	Institution	1.30(1.15,1.47)	Ref.	1.43(1.28,1.60)	Ref.	1.26(1.08,1.48)	Ref.
	Home	1.49(1.34,1.66)	0.10	1.63(1.49,1.80)	0.08	1.12(0.97,1.31)	0.29
Birth quarter	Q1	1.38(1.04,1.82)	Ref.	1.54(1.20,1.97)	Ref.	1.12(0.77,1.63)	Ref.
	Q2	1.38(1.10,1.73)	0.99	1.69(1.38,2.07)	0.56	1.05(0.77,1.43)	0.79
	Q3	1.66(1.33,2.08)	0.31	1.85(1.52,2.26)	0.25	1.74(1.29,2.35)	0.07
	Q4	1.79(1.40,2.28)	0.17	1.89(1.52,2.35)	0.22	1.53(1.11,2.10)	0.22
Safe water							
	Yes	1.28(1.10,1.49)	Ref.	1.43(1.25,1.65)	Ref.	1.31(1.08,1.60)	Ref.
	No	1.49(1.36,1.64)	0.09	1.63(1.49,1.77)	0.13	1.18(1.04,1.34)	0.37
Education							
	No education	1.43(1.28,1.60)	Ref.	1.54(1.39,1.70)	Ref.	1.18(1.01,1.37)	Ref.
	Primary	1.57(1.35,1.83)	0.35	1.83(1.61,2.10)	0.04	1.27(1.03,1.57)	0.56
	Secondary or higher	1.17(0.97,1.41)	0.06	1.23(1.03,1.46)	0.03	1.20(0.96,1.51)	0.87
Household wealth							
	Poorest or poorer	1.39(1.24,1.57)	Ref.	1.50(1.35,1.66)	Ref.	1.12(0.95,1.31)	Ref.
	Middle or richer	1.57(1.38,1.79)	0.17	1.76(1.57,1.98)	0.04	1.39(1.17,1.66)	0.07
	Richest	1.21(0.96,1.54)	0.30	1.39(1.12,1.72)	0.53	1.19(0.88,1.62)	0.71
Geographic zone							
	South, Southeast and Western Asia	1.06(0.92,1.23)	Ref.	1.03(0.90,1.19)	Ref.	1.04(0.88,1.24)	Ref.
	Northern and Western Africa	1.23(1.07,1.41)	0.15	1.23(1.09,1.38)	0.06	1.04(0.86,1.26)	0.99
	Central, Eastern and Southern Africa	2.09(1.81,2.41)	<0.001	2.35(2.07,2.65)	<0.001	1.78(1.45,2.19)	<0.001

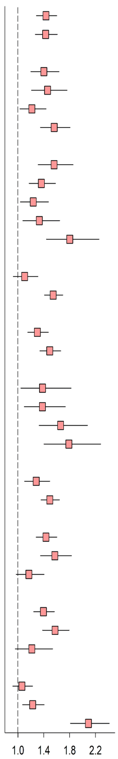

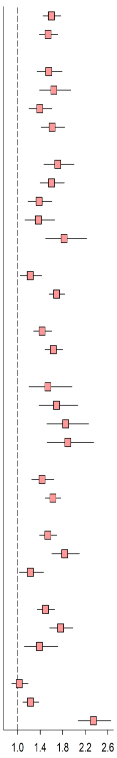

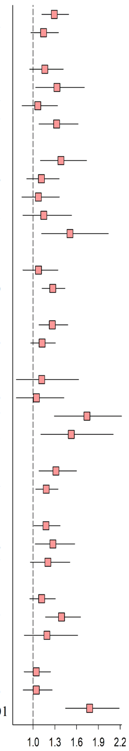

Supplementary Figure S5. Cumulative associations between child mortality and high level of ESPI (90th percentile) at 0-12 preconceptional and prenatal months of mothers stratified by characteristics of study participants. Notes: The sample sizes used to derive statistics are provided in Table 1. The effect estimate of ESPI on child survival computed as the HR of the 90th percentile of ESPI relative to the reference value (defined at 0) is presented as the center of error bars, and the error bars are represent 95% confidence intervals of the estimates. Abbreviations: HR, hazard ratio; CI, confidence interval; ESPI, ENSO precipitation index; ENSO, El Niño Southern Oscillation. The differential association estimates between a subgroup and the reference (Ref.) are tested using the two-sided two-sample z-test given by Equation (1) presented in the main text. Adjustments are not made for multiple comparisons.

Characteristics	Subgroup	Infant mortality HR (95%CI)	Infant mortality Effect modification	Under-five mortality HR (95%CI)	Under-five mortality Effect modification	Neonatal mortality HR (95%CI)	Neonatal mortality Effect modification
Sex							
	Male	1.26(1.16,1.37)	Ref.	1.37(1.27,1.47)	Ref.	1.14(1.03,1.26)	Ref.
	Female	1.36(1.25,1.49)	0.21	1.41(1.30,1.53)	0.54	1.14(1.01,1.28)	0.99
Birth order							
	First	1.21(1.07,1.36)	Ref.	1.34(1.20,1.49)	Ref.	1.01(0.87,1.17)	Ref.
	Second	1.39(1.21,1.59)	0.14	1.50(1.32,1.70)	0.18	1.23(1.03,1.48)	0.09
	Third or fourth	1.18(1.04,1.33)	0.76	1.28(1.15,1.42)	0.56	1.11(0.94,1.30)	0.40
	≥Fifth	1.41(1.27,1.57)	0.05	1.40(1.27,1.54)	0.53	1.22(1.06,1.41)	0.07
Mother age							
	[15, 19)	1.35(1.18,1.54)	Ref.	1.49(1.32,1.68)	Ref.	1.12(0.94,1.33)	Ref.
	[20, 24)	1.29(1.15,1.44)	0.62	1.43(1.29,1.58)	0.62	1.11(0.96,1.28)	0.93
	[25, 29)	1.13(0.99,1.29)	0.07	1.25(1.11,1.40)	0.04	1.06(0.90,1.26)	0.68
	[30, 34)	1.38(1.19,1.61)	0.80	1.29(1.13,1.48)	0.14	1.23(1.01,1.51)	0.47
	≥35	1.48(1.26,1.73)	0.39	1.49(1.29,1.72)	0.97	1.22(0.99,1.50)	0.55
Urbanicity							
	Urban	1.12(0.99,1.27)	Ref.	1.21(1.08,1.35)	Ref.	1.09(0.93,1.27)	Ref.
	Rural	1.38(1.28,1.47)	0.005	1.45(1.37,1.55)	0.005	1.15(1.05,1.26)	0.55
Child delivery place							
	Institution	1.15(1.06,1.26)	Ref.	1.22(1.13,1.32)	Ref.	1.11(0.99,1.24)	Ref.
	Home	1.44(1.32,1.56)	<0.001	1.51(1.40,1.62)	<0.001	1.12(0.99,1.25)	0.99
Birth quarter	Q1	1.86(1.40,2.46)	Ref.	2.24(1.75,2.85)	Ref.	1.47(0.99,2.17)	Ref.
	Q2	1.57(1.02,2.41)	0.52	2.37(1.63,3.44)	0.8	0.96(0.53,1.73)	0.24
	Q3	1.48(1.06,2.08)	0.32	1.93(1.43,2.59)	0.45	1.39(0.88,2.20)	0.86
	Q4	0.92(0.72,1.17)	<0.001	0.92(0.74,1.14)	<0.001	1.02(0.74,1.41)	0.15
Safe water							
	Yes	1.21(1.08,1.35)	Ref.	1.29(1.16,1.43)	Ref.	1.17(1.01,1.35)	Ref.
	No	1.35(1.25,1.45)	0.11	1.42(1.34,1.52)	0.11	1.12(1.02,1.23)	0.62
Education							
	No education	1.33(1.22,1.45)	Ref.	1.39(1.29,1.49)	Ref.	1.13(1.01,1.26)	Ref.
	Primary	1.37(1.22,1.54)	0.66	1.53(1.39,1.70)	0.12	1.10(0.94,1.29)	0.80
	Secondary or higher	1.13(0.99,1.28)	0.03	1.16(1.02,1.31)	0.01	1.14(0.98,1.34)	0.90
Household wealth							
	Poorest or poorer	1.30(1.20,1.41)	Ref.	1.35(1.26,1.46)	Ref.	1.07(0.96,1.2)	Ref.
	Middle or richer	1.36(1.23,1.50)	0.52	1.48(1.35,1.61)	0.14	1.23(1.08,1.4)	0.11
	Richest	1.21(1.02,1.45)	0.48	1.30(1.10,1.52)	0.63	1.13(0.9,1.41)	0.70
Geographic zone							
	South, Southeast and Western Asia	1.19(1.07,1.33)	Ref.	1.18(1.06,1.31)	Ref.	1.1(0.96,1.25)	Ref.
	Northern and Western Africa	1.13(1.03,1.24)	0.09	1.11(1.02,1.20)	0.005	1.03(0.9,1.17)	0.46
	Central, Eastern and Southern Africa	1.65(1.47,1.84)	<0.001	1.80(1.63,1.99)	<0.001	1.4(1.19,1.64)	0.02

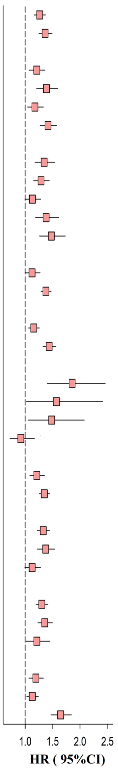

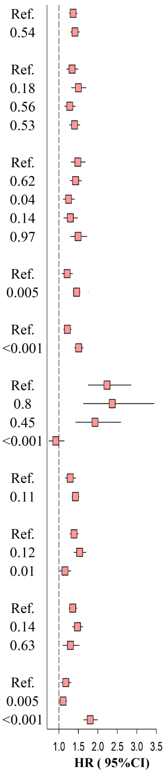

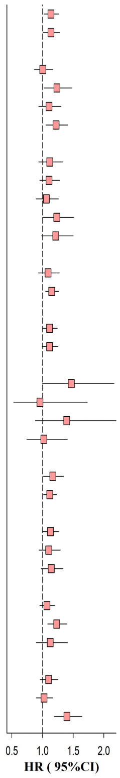

Supplementary Figure S6. Cumulative associations between child mortality and high level of ONI (90th percentile) at 0-12 preconceptional and prenatal months of mothers stratified by characteristics of study participants. Notes: The sample sizes used to derive statistics are provided in Table 1. The effect estimate of ONI on child survival computed as the HR of the 90th percentile of ONI relative to the reference value (defined at 0) is presented as the center of error bars, and the error bars are represent 95% confidence intervals of the estimates. Abbreviations: HR, hazard ratio; CI, confidence interval; ONI, oceanic Niño index. The differential association estimates between a subgroup and the reference (Ref.) are tested using the two-sided two-sample z-test given by Equation (1) presented in the main text. Adjustments are not made for multiple comparisons.

Neonatal mortality and El Niño conditions (based on 90th percentile of MEI measure)

Region	Country	logHR	SE(logHR)	HR	95% CI	Weight
Eastern Africa	Burundi	-0.1432	0.2641	0.87	[0.52; 1.45]	2.7%
Eastern Africa	Kenya	0.3018	0.2109	1.35	[0.89; 2.04]	3.3%
Eastern Africa	Madagascar	2.9459	0.8762	19.03	[3.42; 105.98]	0.4%
Eastern Africa	Malawi	0.2424	0.1227	1.27	[1.00; 1.62]	4.7%
Eastern Africa	Mozambique	1.1178	0.6508	3.06	[0.85; 10.95]	0.7%
Eastern Africa	Rwanda	0.5577	0.1472	1.75	[1.31; 2.33]	4.3%
Eastern Africa	Uganda	0.3764	0.1480	1.46	[1.09; 1.95]	4.3%
Eastern Africa	Tanzania	0.6612	0.1927	1.94	[1.33; 2.83]	3.6%
Western Africa	Benin	-0.0402	0.1610	0.96	[0.70; 1.32]	4.1%
Western Africa	Ghana	-0.0751	0.2474	0.93	[0.57; 1.51]	2.9%
Western Africa	Guinea	0.1740	0.1550	1.19	[0.88; 1.61]	4.2%
Western Africa	Cote dIvoire	-0.1123	0.2844	0.89	[0.51; 1.56]	2.5%
Western Africa	Liberia	0.0791	0.5079	1.08	[0.40; 2.93]	1.1%
Western Africa	Mali	0.1859	0.1115	1.20	[0.97; 1.50]	4.9%
Western Africa	Niger	0.4692	0.4522	1.60	[0.66; 3.88]	1.3%
Western Africa	Nigeria	0.0674	0.1181	1.07	[0.85; 1.35]	4.8%
Western Africa	Senegal	-0.2807	0.1573	0.76	[0.55; 1.03]	4.2%
Western Africa	Burkina Faso	-0.0092	0.1713	0.99	[0.71; 1.39]	3.9%
Southern Africa	Zimbabwe	0.4623	0.2312	1.59	[1.01; 2.50]	3.1%
Southern Africa	Lesotho	0.8014	0.5306	2.23	[0.79; 6.30]	1.0%
Southern Africa	Namibia	-0.6175	0.3345	0.54	[0.28; 1.04]	2.0%
Southern Africa	South Africa	-0.2930	0.5887	0.75	[0.24; 2.36]	0.8%
Northern Africa	Morocco	-0.5270	0.5853	0.59	[0.19; 1.86]	0.8%
Northern Africa	Sudan	0.4991	0.9887	1.65	[0.24; 11.44]	0.3%
Northern Africa	Tunisia	0.4442	1.0688	1.56	[0.19; 12.67]	0.3%
Northern Africa	Egypt	0.5060	0.1395	1.66	[1.26; 2.18]	4.4%
Central Africa	Angola	-2.1288	2.2034	0.12	[0.00; 8.93]	0.1%
Central Africa	Cameroon	-0.0983	0.4537	0.91	[0.37; 2.21]	1.3%
Central Africa	Chad	1.1749	0.3327	3.24	[1.69; 6.22]	2.0%
Central Africa	Congo Democratic Republic	0.7581	0.3443	2.13	[1.09; 4.19]	1.9%
Central Africa	Zambia	0.1862	0.1537	1.20	[0.89; 1.63]	4.2%
South Asia	Bangladesh	0.0912	0.1519	1.10	[0.81; 1.48]	4.2%
South Asia	Sri Lanka	0.3934	1.2678	1.48	[0.12; 17.78]	0.2%
South Asia	India	0.0854	0.0542	1.09	[0.98; 1.21]	5.7%
South Asia	Pakistan	-0.3050	0.1627	0.74	[0.54; 1.01]	4.1%
Southeast Asia	Myanmar	-0.5219	2.2974	0.59	[0.01; 53.57]	0.1%
Western Asia	Jordan	0.3811	0.2029	1.46	[0.98; 2.18]	3.5%
Western Asia	Yemen	-0.0580	0.3281	0.94	[0.50; 1.80]	2.1%
**Random effects model**				**1.21**	**[1.08; 1.36]**	**100.0%**

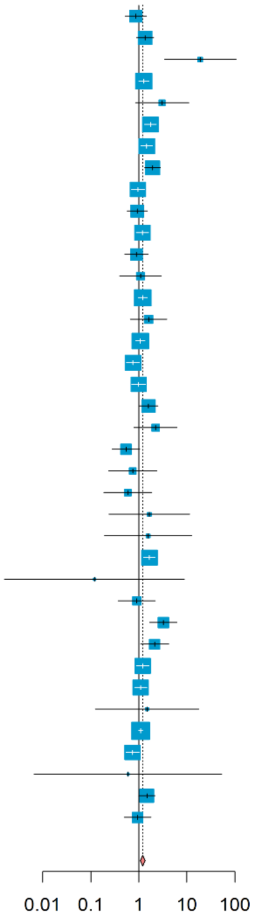

Heterogeneity: $I^2 = 57\%$, $\tau^2 = 0.0566$, $p < 0.01$

Supplementary Figure S7. Cumulative association between neonatal mortality and mothers' exposure to El Niño conditions at 0-12 preconceptional and prenatal months—results from country-specific analyses and meta-analyses. Notes: The country-specific sample sizes used to derive statistics are provided in Supplementary Table S2. The effect estimate, which is presented as the center of the error bars in the figure, is computed as the HR of 90th percentile level of MEI measure relative to the reference value (set at zero). The error bars represent the 95% confidence intervals of estimates. Abbreviations: HR, hazard ratio; CI, confidence interval; MEI, multivariate El Niño index. The heterogeneity is measured by Cochran's Q, which is calculated as the weighted sum of squared differences between country-specific effects and the pooled effect across studies. The Q-statistics is 86.72; with the degree of freedom being 37, the exact *P*-value is 7.19×10^{-6}, which is way smaller than 0.01. Adjustments are not made for multiple comparisons.

Infant mortality and El Niño conditions (based on 90th percentile of MEI measure)

Region	Country	logHR	SE(logHR)	HR	95% CI	Weight
Eastern Africa	Burundi	0.0702	0.2054	1.07	[0.72; 1.60]	3.0%
Eastern Africa	Kenya	0.5832	0.1540	1.79	[1.32; 2.42]	3.4%
Eastern Africa	Madagascar	3.2340	0.6385	25.38	[7.26; 88.72]	0.9%
Eastern Africa	Malawi	0.5193	0.0787	1.68	[1.44; 1.96]	3.9%
Eastern Africa	Mozambique	0.5277	0.4266	1.70	[0.73; 3.91]	1.5%
Eastern Africa	Rwanda	0.6626	0.0982	1.94	[1.60; 2.35]	3.7%
Eastern Africa	Uganda	0.5115	0.0993	1.67	[1.37; 2.03]	3.7%
Eastern Africa	Tanzania	1.0161	0.1251	2.76	[2.16; 3.53]	3.6%
Western Africa	Benin	0.1598	0.1105	1.17	[0.94; 1.46]	3.7%
Western Africa	Ghana	0.1709	0.1840	1.19	[0.83; 1.70]	3.1%
Western Africa	Guinea	0.3009	0.1095	1.35	[1.09; 1.67]	3.7%
Western Africa	Cote dIvoire	-0.0614	0.2180	0.94	[0.61; 1.44]	2.9%
Western Africa	Liberia	-0.4957	0.3762	0.61	[0.29; 1.27]	1.8%
Western Africa	Mali	0.3482	0.0779	1.42	[1.22; 1.65]	3.9%
Western Africa	Niger	0.4680	0.3531	1.60	[0.80; 3.19]	1.9%
Western Africa	Nigeria	0.1144	0.0862	1.12	[0.95; 1.33]	3.8%
Western Africa	Senegal	-0.2685	0.1260	0.76	[0.60; 0.98]	3.6%
Western Africa	Burkina Faso	0.1631	0.1126	1.18	[0.94; 1.47]	3.7%
Southern Africa	Zimbabwe	0.4247	0.1601	1.53	[1.12; 2.09]	3.3%
Southern Africa	Lesotho	1.0097	0.3735	2.74	[1.32; 5.71]	1.8%
Southern Africa	Namibia	-0.4937	0.2350	0.61	[0.39; 0.97]	2.7%
Southern Africa	South Africa	-0.2507	0.5051	0.78	[0.29; 2.09]	1.2%
Northern Africa	Morocco	-0.3645	0.4395	0.69	[0.29; 1.64]	1.5%
Northern Africa	Sudan	0.0945	0.8195	1.10	[0.22; 5.48]	0.6%
Northern Africa	Tunisia	0.1697	0.8274	1.18	[0.23; 6.00]	0.6%
Northern Africa	Egypt	0.5453	0.1036	1.73	[1.41; 2.11]	3.7%
Central Africa	Angola	-1.2705	1.7429	0.28	[0.01; 8.55]	0.1%
Central Africa	Cameroon	0.1361	0.3279	1.15	[0.60; 2.18]	2.1%
Central Africa	Chad	0.9847	0.2277	2.68	[1.71; 4.18]	2.8%
Central Africa	Congo Democratic Republic	0.3723	0.2320	1.45	[0.92; 2.29]	2.7%
Central Africa	Zambia	0.2728	0.1009	1.31	[1.08; 1.60]	3.7%
South Asia	Bangladesh	0.1563	0.1198	1.17	[0.92; 1.48]	3.6%
South Asia	Sri Lanka	1.7623	1.2691	5.83	[0.48; 70.08]	0.3%
South Asia	India	0.1055	0.0462	1.11	[1.02; 1.22]	4.0%
South Asia	Pakistan	-0.0896	0.1284	0.91	[0.71; 1.18]	3.5%
Southeast Asia	Myanmar	0.2922	1.8299	1.34	[0.04; 48.37]	0.1%
Western Asia	Jordan	0.3712	0.1676	1.45	[1.04; 2.01]	3.3%
Western Asia	Yemen	-0.2493	0.2412	0.78	[0.49; 1.25]	2.7%
**Random effects model**				**1.34**	**[1.17; 1.53]**	**100.0%**

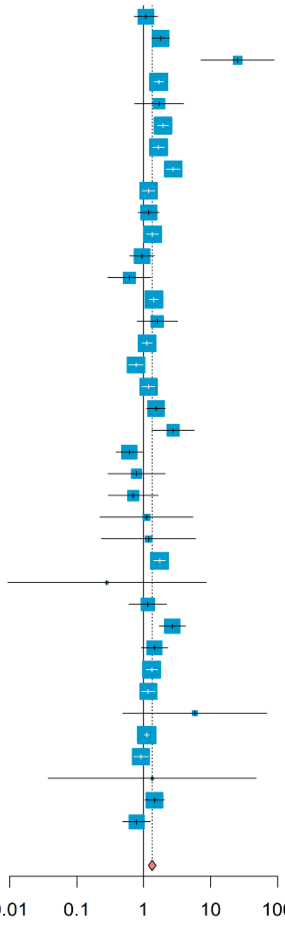

Heterogeneity: $I^2 = 81\%$, $\tau^2 = 0.1120$, $p < 0.01$

Supplementary Figure S8. Cumulative association between infant mortality and mothers' exposure to El Niño conditions at 0-12 preconceptional and prenatal months—results from country-specific analyses and meta-analyses. Notes: The country-specific sample sizes used to derive statistics are provided in Supplementary Table S2. The effect estimate, which is presented as the center of the error bars in the figure, is computed as the HR of 90th percentile level of MEI measure relative to the reference value (set at zero). The error bars represent the 95% confidence intervals of estimates. Abbreviations: HR, hazard ratio; CI, confidence interval; MEI, multivariate El Niño index. The heterogeneity is measured by Cochran's Q, which is calculated as the weighted sum of squared differences between country-specific effects and the pooled effect across studies. The Q-statistics is 190.31; with the degree of freedom being 37, the exact P-value is 1.62×10^{-22}, which is way smaller than 0.01. Adjustments are not made for multiple comparisons.

Neonatal mortality and La Niña conditions (based on 10th percentile of MEI

Region	Country	logHR	SE(logHR)	HR	95% CI	Weight
Eastern Africa	Burundi	0.5878	0.6505	1.80	[0.50; 6.44]	0.7%
Eastern Africa	Kenya	-0.5435	0.1865	0.58	[0.40; 0.84]	3.6%
Eastern Africa	Madagascar	-0.4556	0.2060	0.63	[0.42; 0.95]	3.3%
Eastern Africa	Malawi	-0.0174	0.1781	0.98	[0.69; 1.39]	3.7%
Eastern Africa	Mozambique	0.1514	0.2262	1.16	[0.75; 1.81]	3.0%
Eastern Africa	Rwanda	-0.3336	0.2153	0.72	[0.47; 1.09]	3.2%
Eastern Africa	Uganda	-0.1309	0.2159	0.88	[0.57; 1.34]	3.2%
Eastern Africa	Tanzania	-0.2634	0.2683	0.77	[0.45; 1.30]	2.5%
Western Africa	Benin	-0.6134	0.1874	0.54	[0.38; 0.78]	3.6%
Western Africa	Ghana	0.2932	0.2530	1.34	[0.82; 2.20]	2.7%
Western Africa	Guinea	-0.4711	0.2257	0.62	[0.40; 0.97]	3.0%
Western Africa	Cote dIvoire	-0.5845	0.2574	0.56	[0.34; 0.92]	2.6%
Western Africa	Liberia	0.0110	0.3271	1.01	[0.53; 1.92]	2.0%
Western Africa	Mali	-0.5966	0.1782	0.55	[0.39; 0.78]	3.7%
Western Africa	Niger	-0.4395	0.2207	0.64	[0.42; 0.99]	3.1%
Western Africa	Nigeria	0.0462	0.1025	1.05	[0.86; 1.28]	4.9%
Western Africa	Senegal	-0.4341	0.1394	0.65	[0.49; 0.85]	4.3%
Western Africa	Burkina Faso	-0.0804	0.3993	0.92	[0.42; 2.02]	1.5%
Southern Africa	Zimbabwe	0.5276	0.2584	1.69	[1.02; 2.81]	2.6%
Southern Africa	Lesotho	-0.2142	0.3943	0.81	[0.37; 1.75]	1.5%
Southern Africa	Namibia	-0.1823	0.3409	0.83	[0.43; 1.63]	1.9%
Southern Africa	South Africa	2.5569	2.3213	12.90	[0.14; 1220.01]	0.1%
Northern Africa	Morocco	-0.0917	0.2091	0.91	[0.61; 1.37]	3.2%
Northern Africa	Sudan	-0.0511	0.7828	0.95	[0.20; 4.41]	0.5%
Northern Africa	Tunisia	3.3496	3.0238	28.49	[0.08; 10681.20]	0.0%
Northern Africa	Egypt	-0.1405	0.1835	0.87	[0.61; 1.25]	3.6%
Central Africa	Angola	0.1327	0.7521	1.14	[0.26; 4.99]	0.5%
Central Africa	Cameroon	0.0593	0.3315	1.06	[0.55; 2.03]	1.9%
Central Africa	Chad	-0.4109	0.1700	0.66	[0.48; 0.93]	3.8%
Central Africa	Congo Democratic Republic	-0.3018	0.2198	0.74	[0.48; 1.14]	3.1%
Central Africa	Zambia	-0.2326	0.1874	0.79	[0.55; 1.14]	3.6%
South Asia	Bangladesh	-0.2769	0.1815	0.76	[0.53; 1.08]	3.7%
South Asia	Sri Lanka	-0.5197	1.5648	0.59	[0.03; 12.77]	0.1%
South Asia	India	-0.3038	0.0556	0.74	[0.66; 0.82]	5.6%
South Asia	Pakistan	0.3906	0.1484	1.48	[1.10; 1.98]	4.2%
Southeast Asia	Myanmar	0.5717	1.2538	1.77	[0.15; 20.68]	0.2%
Western Asia	Jordan	0.3918	0.2373	1.48	[0.93; 2.36]	2.9%
Western Asia	Yemen	-0.5201	0.2739	0.59	[0.35; 1.02]	2.5%
**Random effects model**				**0.83**	**[0.74; 0.93]**	**100.0%**

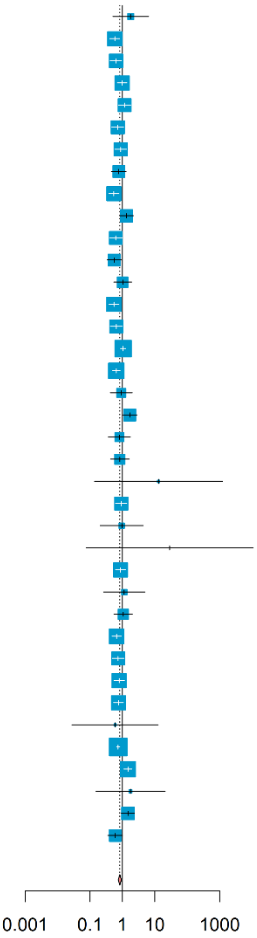

Heterogeneity: $I^2 = 53\%$, $\tau^2 = 0.0533$, $p < 0.01$

Supplementary Figure S9. Cumulative associations between neonatal mortality and mothers' exposure to La Niña conditions at 0-12 preconceptional and prenatal months—results from country-specific analyses and meta-analyses. Notes: The country-specific sample sizes used to derive statistics are provided in Supplementary Table S2. The effect estimate, which is presented as the center of the error bars in the figure, is computed as the HR of 10th percentile level of MEI measure relative to the reference value (set at zero). The error bars represent the 95% confidence intervals of estimates. Abbreviations: HR, hazard ratio; CI, confidence interval; MEI, multivariate El Niño index. The heterogeneity is measured by Cochran's Q, which is calculated as the weighted sum of squared differences between country-specific effects and the pooled effect across studies. The Q-statistics is 78.98; with the degree of freedom being 37, the exact P-value is 7.13×10^{-5}, which is way smaller than 0.01. Adjustments are not made for multiple comparisons.

Infant mortality and La Niña conditions (based on 10th percentile of MEI measure)

Region	Country	logHR	SE(logHR)	HR	95% CI	Weight
Eastern Africa	Burundi	0.1452	0.5223	1.16	[0.42; 3.22]	0.9%
Eastern Africa	Kenya	-0.6321	0.1345	0.53	[0.41; 0.69]	3.5%
Eastern Africa	Madagascar	-0.2743	0.1443	0.76	[0.57; 1.01]	3.4%
Eastern Africa	Malawi	-0.0149	0.1283	0.99	[0.77; 1.27]	3.6%
Eastern Africa	Mozambique	-0.0846	0.1770	0.92	[0.65; 1.30]	3.0%
Eastern Africa	Rwanda	-0.6772	0.1661	0.51	[0.37; 0.70]	3.1%
Eastern Africa	Uganda	-0.0910	0.1530	0.91	[0.68; 1.23]	3.3%
Eastern Africa	Tanzania	-0.4062	0.1960	0.67	[0.45; 0.98]	2.8%
Western Africa	Benin	-0.4119	0.1378	0.66	[0.51; 0.87]	3.5%
Western Africa	Ghana	0.1321	0.2008	1.14	[0.77; 1.69]	2.8%
Western Africa	Guinea	-0.4672	0.1595	0.63	[0.46; 0.86]	3.2%
Western Africa	Cote dlvoire	-0.5979	0.1903	0.55	[0.38; 0.80]	2.9%
Western Africa	Liberia	-0.0385	0.2392	0.96	[0.60; 1.54]	2.4%
Western Africa	Mali	-0.7974	0.1363	0.45	[0.34; 0.59]	3.5%
Western Africa	Niger	-0.7047	0.1552	0.49	[0.36; 0.67]	3.3%
Western Africa	Nigeria	-0.1365	0.0774	0.87	[0.75; 1.02]	4.1%
Western Africa	Senegal	-0.4156	0.1080	0.66	[0.53; 0.82]	3.8%
Western Africa	Burkina Faso	0.2473	0.2662	1.28	[0.76; 2.16]	2.1%
Southern Africa	Zimbabwe	0.0176	0.1931	1.02	[0.70; 1.49]	2.8%
Southern Africa	Lesotho	-0.3980	0.2919	0.67	[0.38; 1.19]	1.9%
Southern Africa	Namibia	-0.1386	0.2420	0.87	[0.54; 1.40]	2.4%
Southern Africa	South Africa	0.7143	1.6546	2.04	[0.08; 52.32]	0.1%
Northern Africa	Morocco	-0.1899	0.1649	0.83	[0.60; 1.14]	3.2%
Northern Africa	Sudan	-0.3840	0.6501	0.68	[0.19; 2.44]	0.6%
Northern Africa	Tunisia	0.1300	2.2600	1.14	[0.01; 95.53]	0.1%
Northern Africa	Egypt	-0.1533	0.1501	0.86	[0.64; 1.15]	3.3%
Central Africa	Angola	0.2264	0.5546	1.25	[0.42; 3.72]	0.8%
Central Africa	Cameroon	0.1642	0.2565	1.18	[0.71; 1.95]	2.2%
Central Africa	Chad	-0.5330	0.1120	0.59	[0.47; 0.73]	3.7%
Central Africa	Congo Democratic Republic	-0.6049	0.1499	0.55	[0.41; 0.73]	3.3%
Central Africa	Zambia	-0.4763	0.1291	0.62	[0.48; 0.80]	3.6%
South Asia	Bangladesh	-0.2776	0.1611	0.76	[0.55; 1.04]	3.2%
South Asia	Sri Lanka	1.3215	1.4125	3.75	[0.24; 59.74]	0.1%
South Asia	India	-0.3484	0.0468	0.71	[0.64; 0.77]	4.3%
South Asia	Pakistan	0.2001	0.1246	1.22	[0.96; 1.56]	3.6%
Southeast Asia	Myanmar	0.5738	0.9727	1.77	[0.26; 11.94]	0.3%
Western Asia	Jordan	0.3124	0.2031	1.37	[0.92; 2.04]	2.7%
Western Asia	Yemen	-0.9078	0.2140	0.40	[0.27; 0.61]	2.6%
**Random effects model**				**0.75**	**[0.68; 0.84]**	**100.0%**

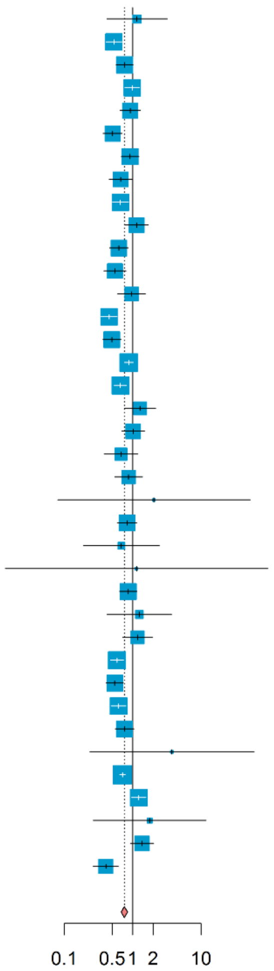

Heterogeneity: $I^2 = 68\%$, $\tau^2 = 0.0658$, $p < 0.01$

Supplementary Figure S10. Cumulative associations between infant mortality and mothers' exposure to La Niña conditions at 0-12 preconceptional and prenatal months—results from country-specific analyses and meta-analyses. Notes: The country-specific sample sizes used to derive statistics are provided in Supplementary Table S2. The effect estimate, which is presented as the center of the error bars in the figure, is computed as the HR of 10th percentile level of MEI measure relative to the reference value (set at zero). The error bars represent the 95% confidence intervals of estimates. Abbreviations: HR, hazard ratio; CI, confidence interval; MEI, multivariate El Niño index. The heterogeneity is measured by Cochran's Q, which is calculated as the weighted sum of squared differences between country-specific effects and the pooled effect across studies. The Q-statistics is 116.40; with the degree of freedom being 37, the exact P-value is 3.85×10^{-10}, which is way smaller than 0.01. Adjustments are not made for multiple comparisons.

Under-five mortality and La Niña conditions (based on 10th percentile of MEI measure)

Region	Country	logHR	SE(logHR)	HR	95% CI	Weight
Eastern Africa	Burundi	-0.4405	0.4802	0.64	[0.25; 1.65]	1.2%
Eastern Africa	Kenya	-0.6837	0.1193	0.50	[0.40; 0.64]	3.3%
Eastern Africa	Madagascar	-0.1665	0.1244	0.85	[0.66; 1.08]	3.3%
Eastern Africa	Malawi	-0.3218	0.1124	0.72	[0.58; 0.90]	3.3%
Eastern Africa	Mozambique	-0.0603	0.1668	0.94	[0.68; 1.31]	3.0%
Eastern Africa	Rwanda	-0.7624	0.1451	0.47	[0.35; 0.62]	3.1%
Eastern Africa	Uganda	-0.1471	0.1405	0.86	[0.66; 1.14]	3.2%
Eastern Africa	Tanzania	-0.3753	0.1668	0.69	[0.50; 0.95]	3.0%
Western Africa	Benin	-0.4971	0.1326	0.61	[0.47; 0.79]	3.2%
Western Africa	Ghana	-0.1751	0.1788	0.84	[0.59; 1.19]	2.9%
Western Africa	Guinea	-0.4939	0.1461	0.61	[0.46; 0.81]	3.1%
Western Africa	Cote dIvoire	-0.6794	0.1823	0.51	[0.35; 0.72]	2.9%
Western Africa	Liberia	-0.0316	0.2064	0.97	[0.65; 1.45]	2.7%
Western Africa	Mali	-1.2105	0.1281	0.30	[0.23; 0.38]	3.3%
Western Africa	Niger	-0.7916	0.1406	0.45	[0.34; 0.60]	3.2%
Western Africa	Nigeria	-0.3288	0.0658	0.72	[0.63; 0.82]	3.6%
Western Africa	Senegal	-0.4737	0.0950	0.62	[0.52; 0.75]	3.4%
Western Africa	Burkina Faso	0.8913	0.2047	2.44	[1.63; 3.64]	2.7%
Southern Africa	Zimbabwe	-0.1404	0.1723	0.87	[0.62; 1.22]	3.0%
Southern Africa	Lesotho	-0.3200	0.2574	0.73	[0.44; 1.20]	2.4%
Southern Africa	Namibia	-0.3461	0.2216	0.71	[0.46; 1.09]	2.6%
Southern Africa	South Africa	1.1664	1.5530	3.21	[0.15; 67.38]	0.2%
Northern Africa	Morocco	-0.1627	0.1577	0.85	[0.62; 1.16]	3.1%
Northern Africa	Sudan	-0.5048	0.5764	0.60	[0.20; 1.87]	1.0%
Northern Africa	Tunisia	-0.2276	2.2044	0.80	[0.01; 59.91]	0.1%
Northern Africa	Egypt	-0.1902	0.1430	0.83	[0.62; 1.09]	3.2%
Central Africa	Angola	0.4401	0.4535	1.55	[0.64; 3.78]	1.3%
Central Africa	Cameroon	-0.0346	0.2331	0.97	[0.61; 1.53]	2.5%
Central Africa	Chad	-0.6155	0.0943	0.54	[0.45; 0.65]	3.4%
Central Africa	Congo Democratic Republic	-0.6354	0.1277	0.53	[0.41; 0.68]	3.3%
Central Africa	Zambia	-0.3898	0.1096	0.68	[0.55; 0.84]	3.4%
South Asia	Bangladesh	-0.2442	0.1571	0.78	[0.58; 1.07]	3.1%
South Asia	Sri Lanka	0.9828	1.3306	2.67	[0.20; 36.26]	0.2%
South Asia	India	-0.3412	0.0439	0.71	[0.65; 0.77]	3.7%
South Asia	Pakistan	0.1841	0.1205	1.20	[0.95; 1.52]	3.3%
Southeast Asia	Myanmar	0.2851	0.9364	1.33	[0.21; 8.33]	0.4%
Western Asia	Jordan	0.2632	0.1970	1.30	[0.88; 1.91]	2.8%
Western Asia	Yemen	-0.9409	0.2047	0.39	[0.26; 0.58]	2.7%
**Random effects model**				**0.72**	**[0.64; 0.82]**	**100.0%**

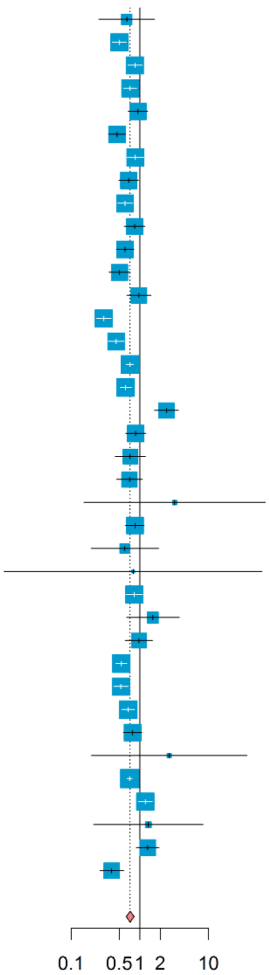

Heterogeneity: $I^2 = 80\%$, $\tau^2 = 0.1161$, $p < 0.01$

Supplementary Figure S11. Cumulative associations between under-five mortality and mothers' exposure to La Niña conditions at 0-12 preconceptional and prenatal months—results from country-specific analyses and meta-analyses. Notes: The country-specific sample sizes used to derive statistics are provided in Supplementary Table S2. The effect estimate, which is presented as the center of the error bars in the figure, is computed as the HR of 10th percentile level of MEI measure relative to the reference value (set at zero). The error bars represent the 95% confidence intervals of estimates. Abbreviations: HR, hazard ratio; CI, confidence interval; MEI, multivariate El Niño index. The heterogeneity is measured by Cochran's Q, which is calculated as the weighted sum of squared differences between country-specific effects and the pooled effect across studies. The Q-statistics is 187.49; with the degree of freedom being 37, the exact *P*-value is 5.12×10^{-22}, which is way smaller than 0.01. Adjustments are not made for multiple comparisons.

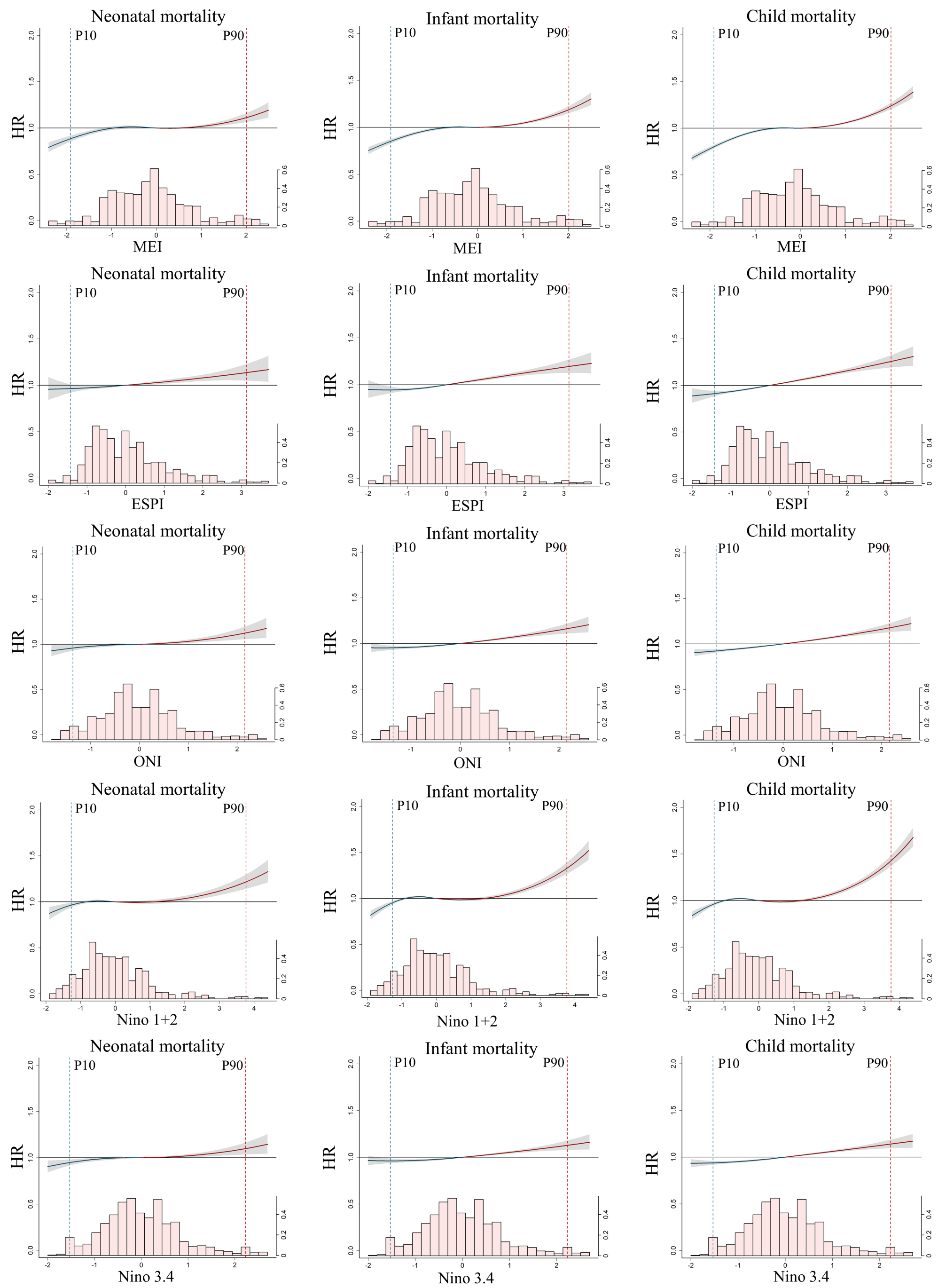

Supplementary Figure S12. Cumulative exposure-response associations between child survival and ENSO during the 9th to 12th lagged (preconceptional) months before mothers' delivery. Notes: The red and blue solid lines (with 95% confidence interval, shaded grey) indicate effect estimates of El Niño-like and La Niña-like conditions, respectively. They are the centers for the error bands. The association estimate of each ENSO measure with child survival is computed as the HR of a given percentile of ENSO relative to the reference value (set at zero). Histograms of ENSO indices are plotted at the bottom, with density measured by the second (right) vertical axis. Abbreviations: HR, hazard ratio; ENSO, El Niño Southern Oscillation; MEI, multivariate El Niño index; ESPI, ENSO precipitation index; ONI, oceanic Niño index; P90, 90th percentile; P10, 10th percentile.

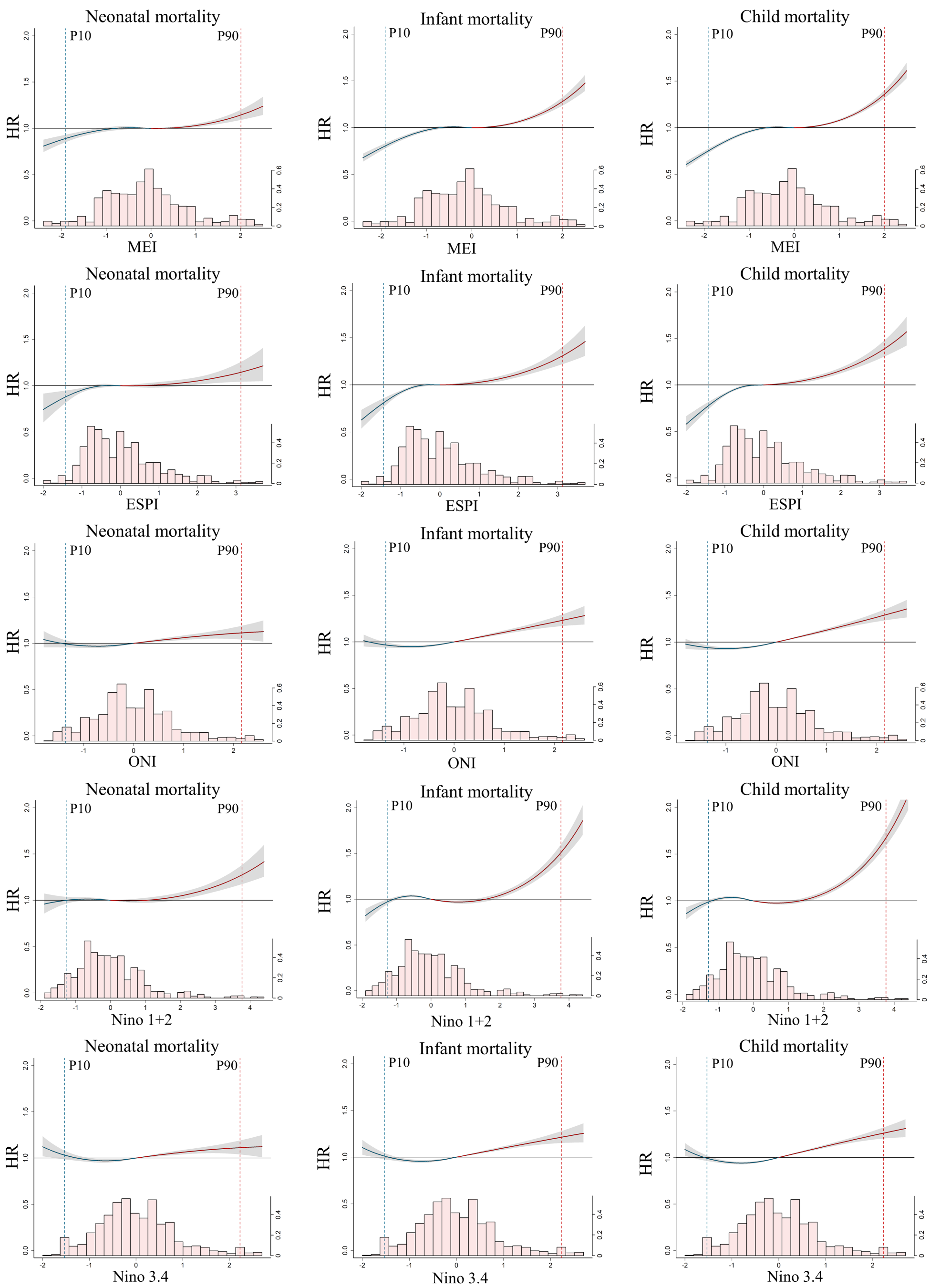

Supplementary Figure S13. Cumulative exposure-response associations between child survival and ENSO during the 0th to 8th lagged (prenatal) months before mothers' delivery. Notes: The red and blue solid lines (with 95% confidence interval, shaded grey) indicate effect estimates of El Niño-like and La Niña-like conditions, respectively. They are the centers for the error bands. The association estimate of each ENSO measure with child survival is computed as the HR of a given percentile of ENSO relative to the reference value (set at zero). Histograms of ENSO indices are plotted at the bottom, with density measured by the second (right) vertical axis. Abbreviations: HR, hazard ratio; ENSO, El Niño Southern Oscillation; MEI, multivariate El Niño index; ESPI, ENSO precipitation index; ONI, oceanic Niño index; P90, 90th percentile; P10, 10th percentile.

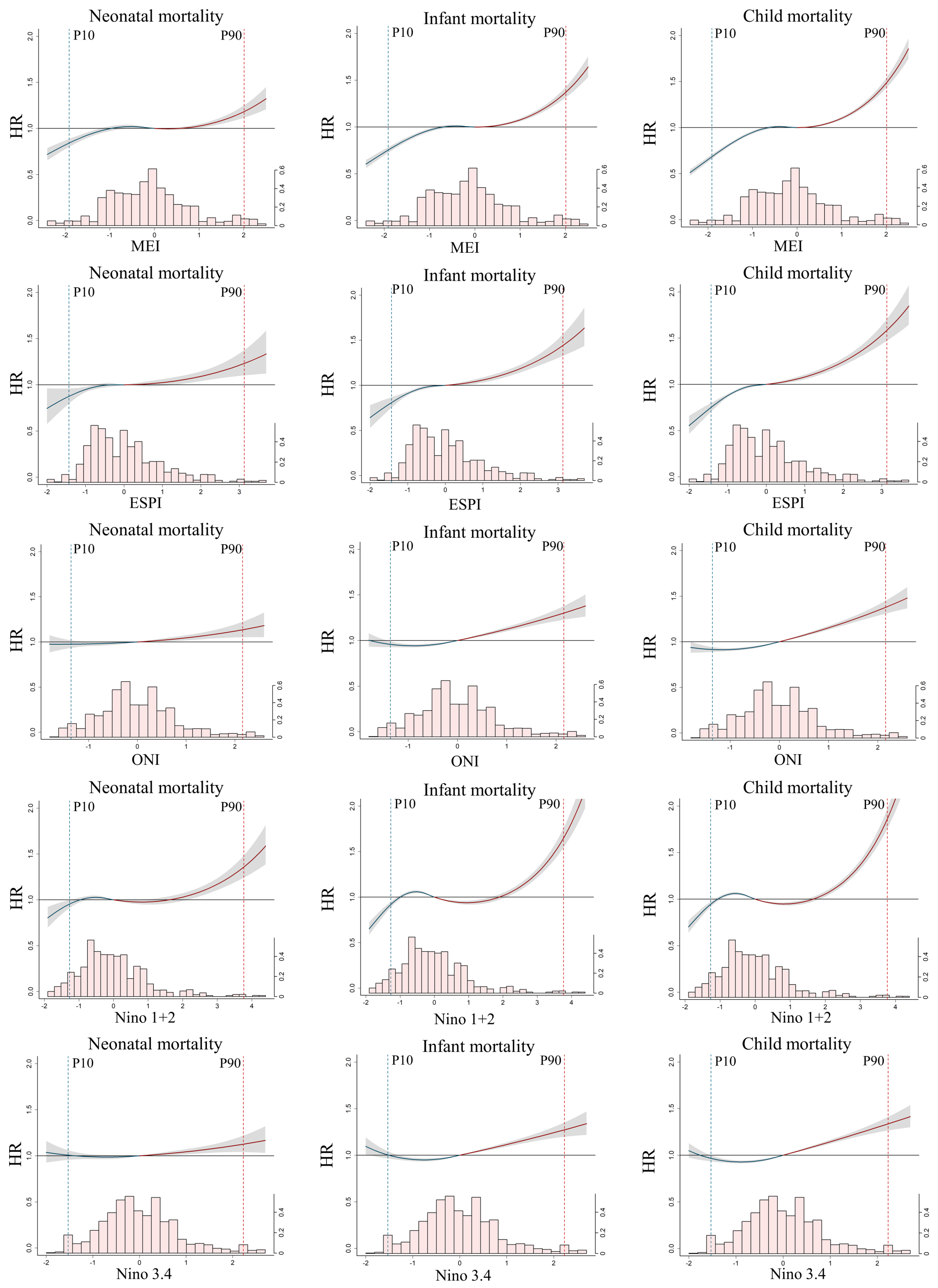

Supplementary Figure S14. Results for sensitivity analysis of cumulative exposure-response associations between child survival and ENSO at lag 0-12 months of mothers' delivery by including a random effect for country variable. Notes: The red and blue solid lines (with 95% confidence interval, shaded grey) indicate effect estimates of El Niño-like and La Niña-like conditions, respectively. They are the centers for the error bands. The association estimate of each ENSO measure with child survival is computed as the HR of a given percentile of ENSO relative to the reference value (set at zero). Histograms of ENSO indices are plotted at the bottom, with density measured by the second (right) vertical axis. Abbreviations: HR, hazard ratio; ENSO, El Niño Southern Oscillation; MEI, multivariate El Niño index; ESPI, ENSO precipitation index; ONI, oceanic Niño index; P90, 90th percentile; P10, 10th percentile.

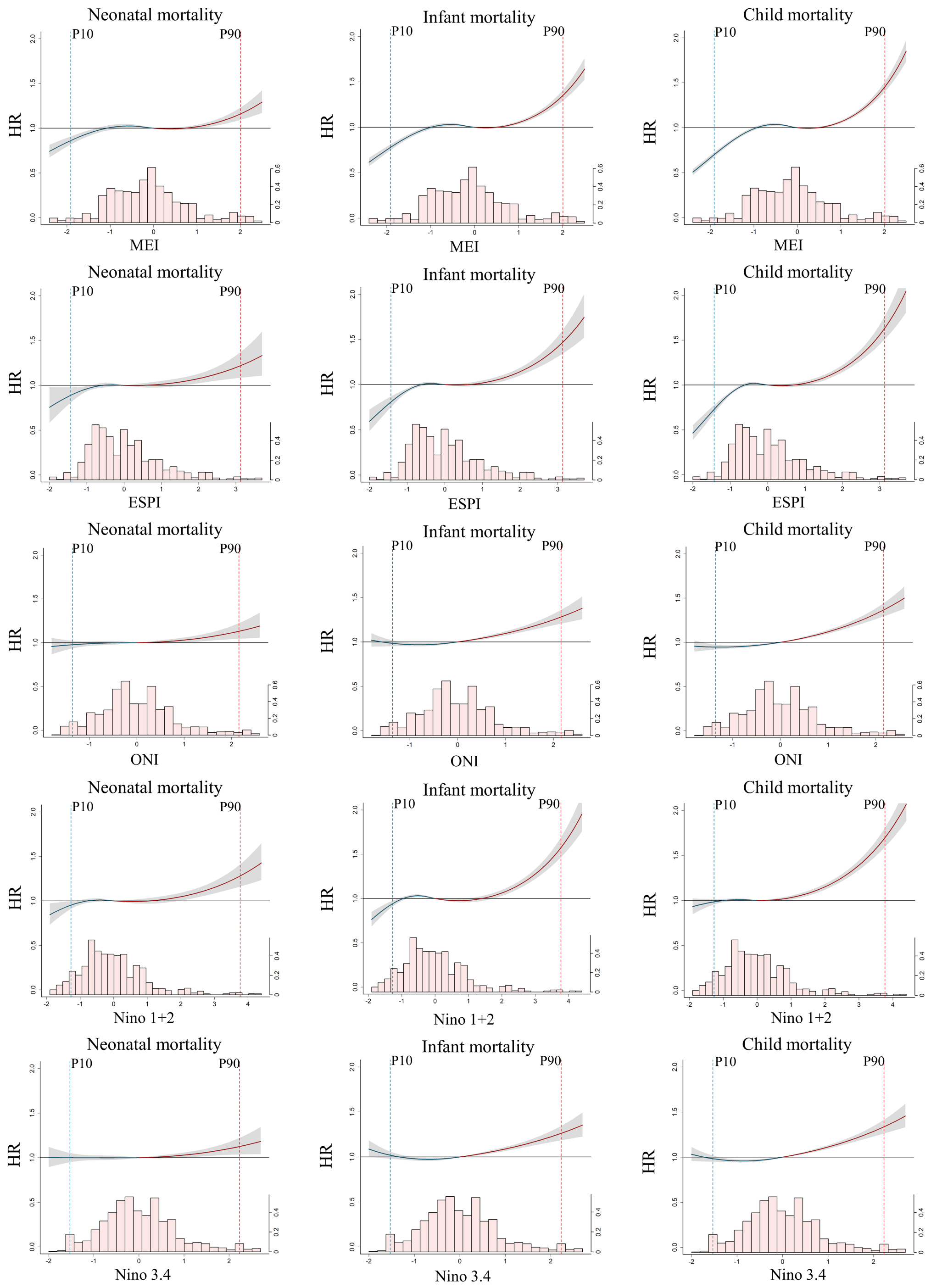

Supplementary Figure S15. Results for sensitivity analysis of cumulative exposure-response associations between child survival and ENSO at lag 0-12 months of mothers' delivery by including the random effect for survey cluster. Notes: The red and blue solid lines (with 95% confidence interval, shaded grey) indicate effect estimates of El Niño-like and La Niña-like conditions, respectively. They are the centers for the error bands. The association estimate of each ENSO measure with child survival is computed as the HR of a given percentile of ENSO relative to the reference value (set at zero). Histograms of ENSO indices are plotted at the bottom, with density measured by the second (right) vertical axis. Abbreviations: HR, hazard ratio; ENSO, El Niño Southern Oscillation; MEI, multivariate El Niño index; ESPI, ENSO precipitation index; ONI, oceanic Niño index; P90, 90th percentile; P10, 10th percentile.

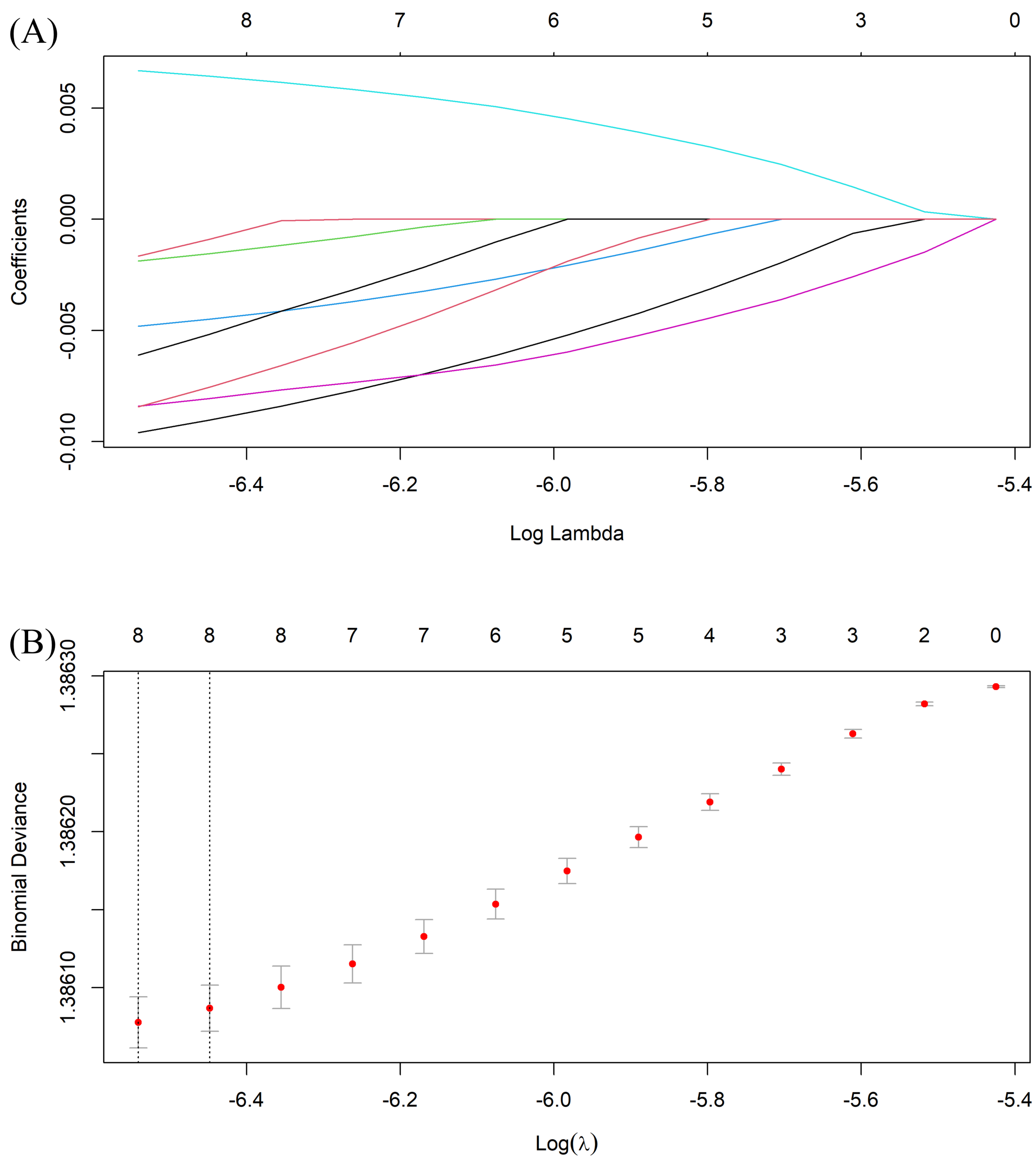

Supplementary Figure S16. Selection of covariates by the LASSO approach for neonatal mortality. Notes: Panel (A) shows the fitting process of λ; panel (B) is the plot of cross-validation curve. The non-zero coefficients were seven covariates, which represented in the sparse matrix format and were identified as the selection of covariates, including child sex, child delivery location, birth order, maternal age at child birth, educational level of mother, access to toilets in the households, and access to safe water.

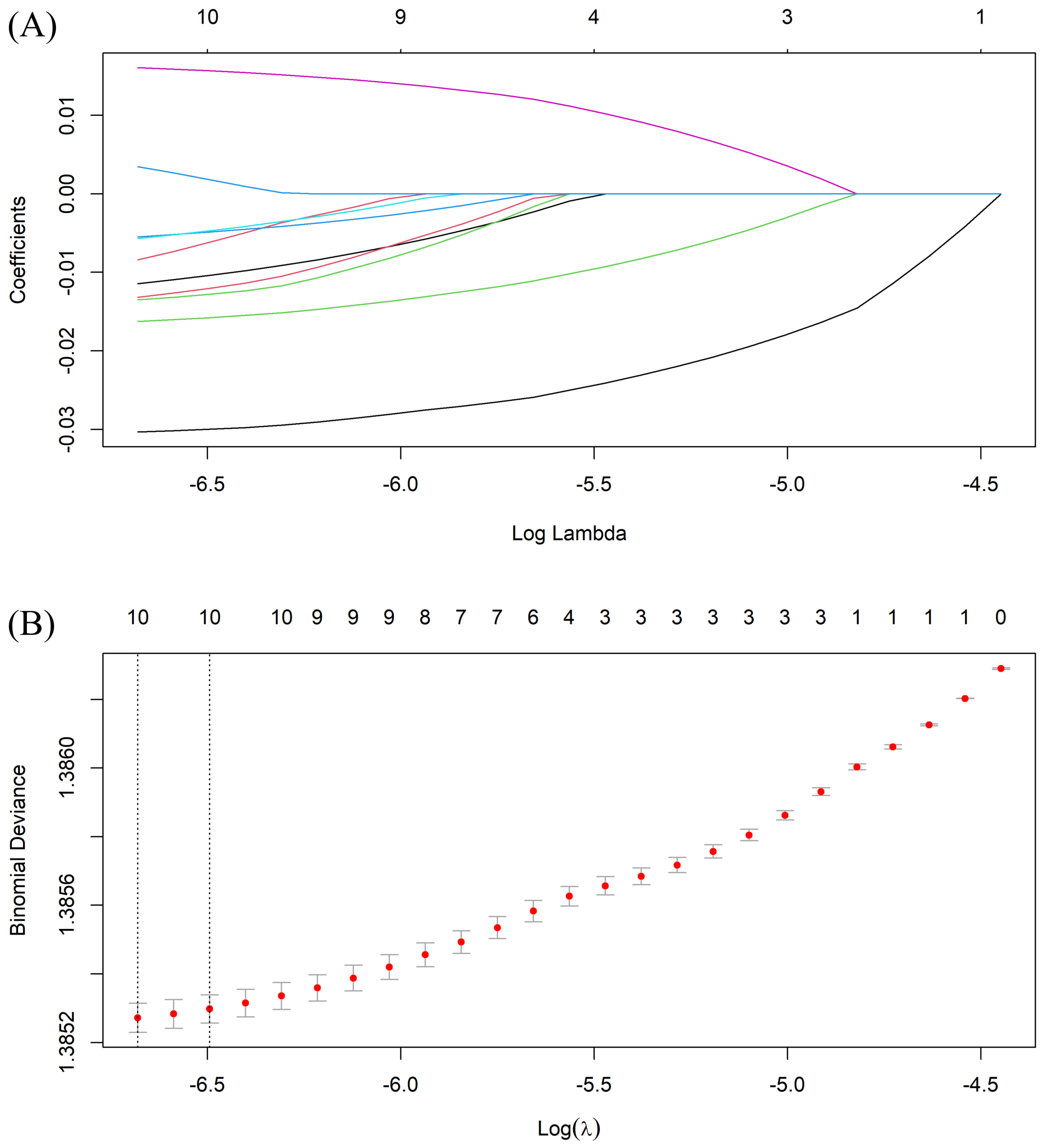

Supplementary Figure S17. Selection of covariates by the LASSO approach for infant mortality. Notes: Panel (A) shows the fitting process of λ; panel (B) is the plot of cross-validation curve. The non-zero coefficients were eight covariates, which represented in the sparse matrix format and were identified as the selection of covariates, including child sex, child delivery location, birth order, maternal age at child birth, maternal marital status, educational level of mother, access to toilets in the households, and access to safe water.

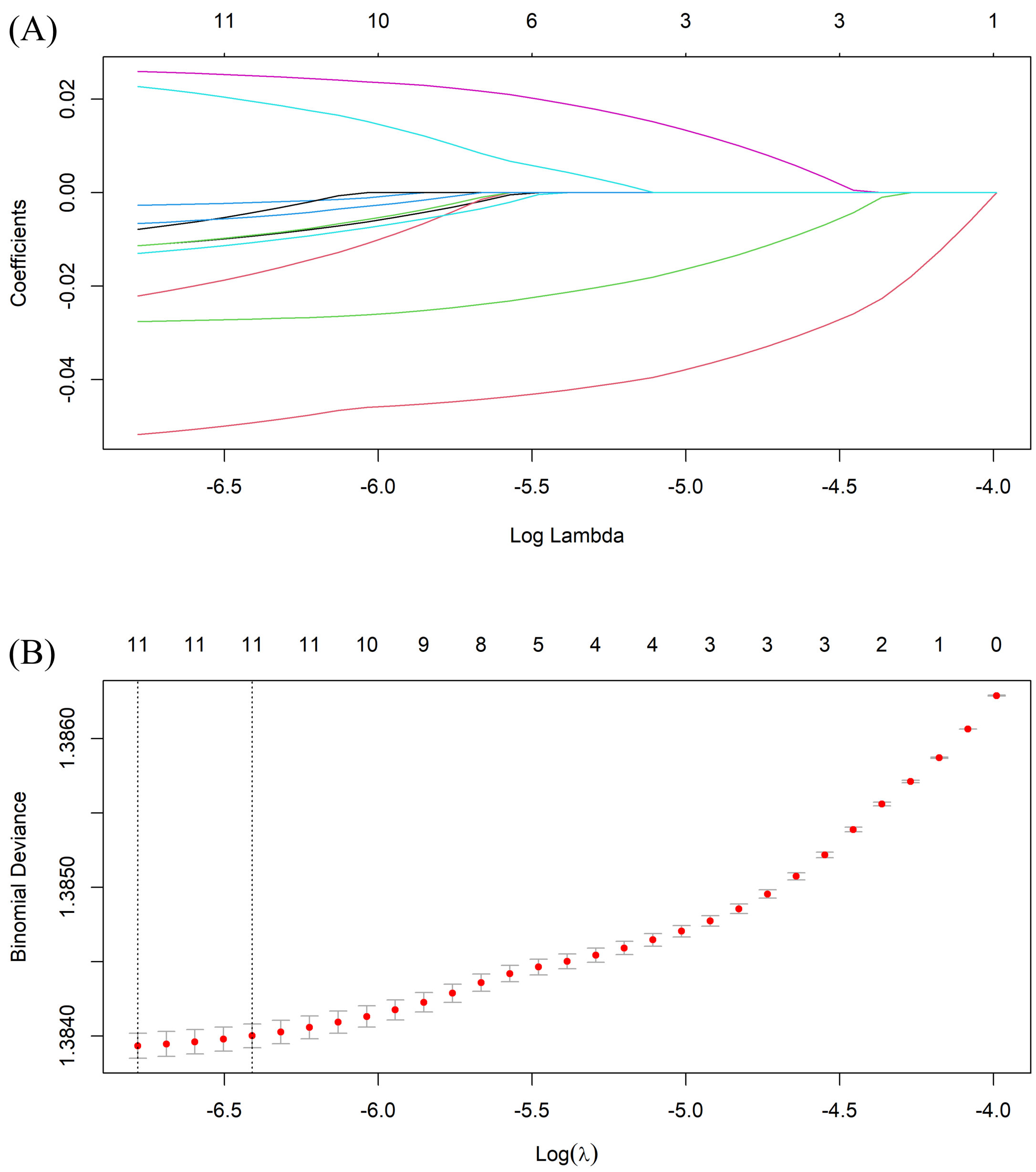

Supplementary Figure S18. Selection of covariates by the LASSO approach for under-five mortality. Notes: Panel (A) shows the fitting process of λ; panel (B) is the plot of cross-validation curve. The non-zero coefficients were weight covariates, which represented in the sparse matrix format and were identified as the selection of covariates, including child sex, child delivery location, birth order, maternal age at child birth, maternal marital status, educational level of mother, access to toilets in the households, and access to safe water.
