# Supplementary material for: Maternal preconceptional and prenatal exposure to El Niño Southern Oscillation levels and child mortality: a multi-country study
Source: Nat Commun. 2024 Jul 17;15:6034. doi: 10.1038/s41467-024-50467-x (PMC11254917; doi:10.1038/s41467-024-50467-x)
Supplement: Supplementary file 3 — Reporting Summary [file 41467_2024_50467_MOESM3_ESM.pdf]

nature portfolio

Corresponding author(s): Castiel Chen Zhuang

Last updated by author(s): Jun 26, 2024

Reporting Summary

Nature Portfolio wishes to improve the reproducibility of the work that we publish. This form provides structure for consistency and transparency in reporting. For further information on Nature Portfolio policies, see our Editorial Policies and the Editorial Policy Checklist.

Statistics

For all statistical analyses, confirm that the following items are present in the figure legend, table legend, main text, or Methods section.

n/a	Confirmed	
☐	☒	The exact sample size (n) for each experimental group/condition, given as a discrete number and unit of measurement
☐	☒	A statement on whether measurements were taken from distinct samples or whether the same sample was measured repeatedly
☐	☒	The statistical test(s) used AND whether they are one- or two-sided<br>*Only common tests should be described solely by name; describe more complex techniques in the Methods section.*
☐	☒	A description of all covariates tested
☐	☒	A description of any assumptions or corrections, such as tests of normality and adjustment for multiple comparisons
☐	☒	A full description of the statistical parameters including central tendency (e.g. means) or other basic estimates (e.g. regression coefficient) AND variation (e.g. standard deviation) or associated estimates of uncertainty (e.g. confidence intervals)
☐	☒	For null hypothesis testing, the test statistic (e.g. F, t, r) with confidence intervals, effect sizes, degrees of freedom and P value noted<br>*Give P values as exact values whenever suitable.*
☒	☐	For Bayesian analysis, information on the choice of priors and Markov chain Monte Carlo settings
☒	☐	For hierarchical and complex designs, identification of the appropriate level for tests and full reporting of outcomes
☐	☒	Estimates of effect sizes (e.g. Cohen's d, Pearson's r), indicating how they were calculated

Our web collection on statistics for biologists contains articles on many of the points above.

Software and code

Policy information about availability of computer code

Data collection	In this study, we included all the publicly available standard surveys facilitated by IPUMS-DHS before October 15, 2022 after excluding countries that did not record calendar dates for interview to avoid comparability issues. IPUMS is an online data dissemination tool (https://www.idhsdata.org/idhs/) that has harmonized various variable names and codes across many DHS samples, making it easier to compile the relevant information on different types of data and perform comparative research comprehensively.
Data analysis	All data analyses were conducted between October 2022 and March 2024 and analyzed using R, version 4.3.3 (R Project for Statistical Computing), and the following packages were primarily used: package “survival” was installed to characterize associations with outcome measures; package “glmnet” was loaded to select variables by LASSO; package “dlnm” was applied to specify the cross-basis function for ENSO exposure and to predict and graphically plot the results of the fitted regression models. The primary R codes are available at https://github.com/castielzhuang/ENSO or https://doi.org/10.5281/zenodo.11408167.

For manuscripts utilizing custom algorithms or software that are central to the research but not yet described in published literature, software must be made available to editors and reviewers. We strongly encourage code deposition in a community repository (e.g. GitHub). See the Nature Portfolio guidelines for submitting code & software for further information.

Data

Policy information about availability of data

All manuscripts must include a data availability statement. This statement should provide the following information, where applicable:

- Accession codes, unique identifiers, or web links for publicly available datasets
- A description of any restrictions on data availability
- For clinical datasets or third party data, please ensure that the statement adheres to our policy

The DHS data used in this study were de-identified and freely available from the program website (https://dhsprogram.com/), but third-party restrictions apply to the availability of the data. The data were used under license for this study with restrictions that do not allow for the data to be redistributed or made publicly available. To gain access to the health and meteorologic data, researchers should register a user account online, provide a brief description of the proposed projects, and receive the approval from the DHS Program at the IPUMS-DHS website (https://www.idhsdata.org/idhs/). This process can be done within a day without data-sharing agreements. The data on the monthly intensity of all the ENSO measures used in this study can be downloaded from the NOAA website at https://psl.noaa.gov/data/climateindices. The GIS boundary files used to draw the maps presented in Figure 1a-c are publicly available from the IPUMS-International website (https://international.ipums.org/international/gis.shtml). Data supporting the findings of this study are all available in the article, in the supplementary files, and from the corresponding author upon request. Source data is included with this paper.

Research involving human participants, their data, or biological material

Policy information about studies with human participants or human data. See also policy information about sex, gender (identity/presentation), and sexual orientation and race, ethnicity and racism.

Reporting on sex and gender	Findings apply to both sexes of children but only to mothers rather than fathers. The study was designed for mothers. Child sex was determined by self-reporting. In our study sample, 765,172 (51.0%) were male and 734,555 (49.0%) were female Sex-based analyses were performed in subgroup analyses, as shown in Figure 4.
Reporting on race, ethnicity, or other socially relevant groupings	The socially relevant categorization variables used in the manuscript include age group, education level, and household wealth index in quintiles. Age and education were self-reported by respondents. The wealth index, generated with a statistical procedure known as principal components analysis, places individual households on a continuous scale of relative wealth. DHS separates all interviewed households into five quintiles of wealth. These socially relevant groupings were treated as covariates in the main regressions.
Population characteristics	Of all the observations in the sample, nearly 47.0% had mothers younger than 25 years old, 70.2% lived in rural areas, 68.8% had mothers with a primary educational level or lower, and 62.4% did not have safe sources of drinking water.
Recruitment	Participants were recruited by DHS. It must be acknowledged that the study sample may not be representative of some conflict-affected areas in countries experiencing wars or insecurity, or countries that opt out of participating in the DHS. The magnitude of this under-representativeness of conflict affected areas may vary from one country to another. For instance, in the Democratic Republic of the Congo (or Congo Democratic Republic), only 4 of the 540 sampled clusters were not accessible due to insecurity in the 2012/2014 DHS surveys (that is, less than 1% all sampled clusters were affected). Therefore, we speculated that conflict conditions were not likely to have substantial influences on the magnitude and direction of the associations observed in this study. Noteworthy was that this speculation was derived based only on the example of the Democratic Republic of Congo, given that we were unable to obtain comparable data for other countries.
Ethics oversight	The data were anonymized or de-identified. Thus, since the study concerns non-human subject research, consent from participants was waived and IRB approval was not required for this study.

Note that full information on the approval of the study protocol must also be provided in the manuscript.

Field-specific reporting

Please select the one below that is the best fit for your research. If you are not sure, read the appropriate sections before making your selection.

☐ Life sciences ☐ Behavioural & social sciences ☒ Ecological, evolutionary & environmental sciences

For a reference copy of the document with all sections, see nature.com/documents/nr-reporting-summary-flat.pdf

Ecological, evolutionary & environmental sciences study design

All studies must disclose on these points even when the disclosure is negative.

Study description	In this paper, we constructed a retrospective cohort of children under-five years of age by pooling all available mortality data from 160 nationally representative Demographic and Health Surveys (DHS).
Research sample	The raw sample consisted of 1,678,065 (see Extended Data Table S3) children under five years of age from 38 low- and middle-income countries, which was used in our meta-analysis; after excluding individuals with missing data, our primary analytic sample consisted of 1,499,727 children under five years of age born between 1981 and 2018 across 34 countries based on all available data.

Sampling strategy	The original sample sizes of each country were determined by the DHS. To enhance international comparability of reproductive-age women, we restricted the analytic sample to children whose mothers were 15 to 49 years old when giving birth, which excluded about 0.4% of the original sample. We further dropped any child born more than five years preceding or after the first day of the mother's interview, which accounted for less than 0.1% of the original sample. These led us to the raw sample used in our meta-analysis.
Data collection	We included all the publicly available standard surveys facilitated by IPUMS-DHS before October 15, 2022.
Timing and spatial scale	Between 1981 and 2018 across 38 low- and middle-income countries in Africa and Asia.
Data exclusions	We excluded children whose mothers were younger than 15 or older than 49 years old when giving birth and any child born more than five years preceding or after the first day of the mother's interview in all analyses, and we further excluded individuals with missing data in the primary analysis.
Reproducibility	The replication codes were run in different computers of the authors, and they were provided publicly (https://github.com/castielzhuang/ENSO).
Randomization	Randomization was not relevant to our study, as this is a retrospective observational study.
Blinding	Blinding was not relevant to our study, as this is a retrospective observational study.

Did the study involve field work? ☐ Yes ☒ No

Reporting for specific materials, systems and methods

We require information from authors about some types of materials, experimental systems and methods used in many studies. Here, indicate whether each material, system or method listed is relevant to your study. If you are not sure if a list item applies to your research, read the appropriate section before selecting a response.

Materials & experimental systems

n/a	Involved in the study	
☒	☐	Antibodies
☒	☐	Eukaryotic cell lines
☒	☐	Palaeontology and archaeology
☒	☐	Animals and other organisms
☒	☐	Clinical data
☒	☐	Dual use research of concern
☒	☐	Plants

Methods

n/a	Involved in the study	
☒	☐	ChIP-seq
☒	☐	Flow cytometry
☒	☐	MRI-based neuroimaging

Plants

Seed stocks	Not applicable.
Novel plant genotypes	Not applicable.
Authentication	Not applicable.
